# Analysis of Polyphenols from *Polygala major* Jacq.

**DOI:** 10.3390/antiox14020153

**Published:** 2025-01-27

**Authors:** Semra Yılmazer Keskin, Ayşe Avcı, Lana Arif Ali Ali, Can Serkan Keskin

**Affiliations:** 1 Department of Chemistry, Faculty of Science, Sakarya University, Sakarya 54050, Turkey; ckeskin@sakarya.edu.tr; 2 Department of Food Engineering, Faculty of Engineering, Sakarya University, Sakarya 54050, Turkey; aysea@sakarya.edu.tr; 3 Institute of Science, Sakarya University, Sakarya 54050, Turkey; lanabayatli@gmail.com

**Keywords:** *Polygala major* Jacq., phenolic compounds, flavonoids, FTIR, LC-MS

## Abstract

Plant polyphenols have significant importance due to their potential health benefits. *Polygala major* Jacq. is commonly found in Asia and has a long history of use in traditional medicine. This study investigated the extraction of polyphenols from the leaves and flowers of *P. major* Jacq. using various solvents (acetone, ethanol, and methanol) and employing two extraction methods: maceration and ultrasonication. Significant amounts of total phenolics were detected in all conditions, measuring between 26.69 and 48.51 mg GAE/g dry matter, with the highest concentration found in the ultrasonicated ethanol extract from the leaves. Compared to the other solvents, acetone was particularly effective in extracting flavonoids from the leaves. The antioxidant activities ranged from 0.65 to 0.86 mg TE/g dry matter, as determined by the DPPH radical scavenging activity method. The leaf extract displayed antibacterial activity against *Bacillus cereus*, *Pseudomonas aeruginosa*, and *Staphylococcus aureus*, while the flower extract had no antibacterial activity. UV-Vis spectroscopy and FTIR analysis confirmed the presence of polyphenols in the extracts. According to LCMS analyses, a total of 67 compounds were identified in the leaves and flowers, including several that are pharmaceutically significant, such as eupatorin, sinensetin, acacetin, ombuin, vanillic acid, sinapic acid, apigenin, naringenin, and luteolin.

## 1. Introduction

The genus *Polygala* belongs to the family of *Polygalaceae*, which comprises around 600 species and is widely distributed in Asian countries [1,2]. The members of the genus are highly diverse, including herbs, shrubs, small trees, and some climbers. Among the genus, 20 species were reported in Turkey, including *Polygala major* Jacq. [3]. The species contain various secondary metabolites, including xanthones, saponins, flavonoids, coumarins, polygalic acid, and styrylpyrones [4,5]. Owing to their richness in metabolites, many species of *Polygala* are used in traditional medicine for various treatments, including inflammatory diseases (stomatitis, myelitis, hepatitis) and refractory diseases (pulmonary tuberculosis, esophageal cancer) [1].

Polyphenols are extracted and separated from plants using various methods. Along with conventional methods, new strategies, such as microwave-assisted extraction, ultrasound-assisted extraction, and supercritical fluid extraction, have been introduced in recent years to maximize extraction efficiency [6]. Among these, ultrasound-assisted extraction has attracted great attention due to its fast, simple, and efficient nature [7]. Ultrasonication works by generating acoustic waves that cause bubbles to cavitate. When these bubbles collapse, they can create physical, chemical, and mechanical effects that can break down the walls of biological cells. Therefore, ultrasonication facilitates the release of extractable compounds and can effectively increase the total amount of phenolics and antioxidant activity in a given sample [8]. A variety of solvents is used for the extraction of phenolic compounds from the plants, including water, organic solvents (acetone, methanol, ethanol, ethyl acetate, n-hexane), and deep eutectic solvents [9,10]. Recent studies have focused on green solvents due to the environmental and health concerns surrounding organic solvents. Water is the most economical and safe solvent, although its extraction efficiency is weak compared to organic solvents [11]. Ethanol is also preferred as an extraction solvent because it is safer than the other solvents such as methanol and acetone [10,12].

Various species of *Polygala* have been investigated for phytoconstituents. Bergeron et al. (1997) reported chromonocoumarins and lignans from *Polygala gazensis, P. teretifolia*, and *P. fruticose* [13]. De Leo et al. (2017) found flavonol glycosides, oligosaccharides, ionone, and triterpenoid saponins from *Polygala flavescens* [14]. Li et al. (2014) determined xanthones from *Polygala glomerata* [15]. Huang et al. (2015) isolated flavonol glycosides from *Polygala sibirica* [16]. Cervellati et al. (2004) extracted flavonoids and biphenyl derivatives from *Polygala alpestris* [17]. Zeleke et al. (2024) screened tannins and saponins from *Polygala sadebeckiana* [18]. Liu et al. (2019) determined terpenoid, xanthone, and oligosaccharide from *Polygala tenuifolia* [19]. However, to the best of our knowledge, studies concerning *P. major* Jacq. are limited. Therefore, this study aimed to investigate the extraction and analysis of phenolic compounds from *P. major* Jacq. using different extraction techniques and solvents.

## 2. Materials and Methods

### 2.1. Materials

*Polygala major* Jacq. was collected (~500 g) from Sakarya University Campus, Esentepe region (40°44′ N, 30°19′ E, 217 m.a.s.l.) in Sakarya in May 2022. At the time of collection, the plant was in the flowering stage. Flowers and leaves were separated and dried at room temperature. Dried samples were mechanically ground and screened through an 18-mesh sieve and stored in plastic bags at room temperature until analysis.

All the chemicals used in the study were of analytical grade. 1,1-Diphenyl-2-picrylhdrazyl (DPPH) was purchased from Sigma-Aldrich (St. Louis, MO, USA), and the others (sodium carbonate, methanol, ethanol, acetone, gallic acid, aluminum chloride, sodium nitrite, sodium hydroxide, quercetin, and Folin-Ciocalteau reagent) were obtained from Merck (Darmstadt, Germany).

### 2.2. Extraction of Polyphenols

The extraction of polyphenols from the flowers and leaves of *P. major* Jacq. was performed using either the maceration technique or ultrasonication. Three different solvents at 80% concentration (acetone, ethanol, and methanol) were used for the extraction. For macerated extraction, 0.5 g of dried samples were mixed with each solvent and agitated at 150 rpm at 30 °C for 24 h. Then, they were centrifuged at 4000 rpm for 15 min, and the supernatant containing phenolic compounds was filtered through Whatman no. 1 filter paper [20]. Ultrasonication-assisted extraction was carried out using the same amount of samples and solvents. An ultrasonication device (Bandelin HD2200, Berlin, Germany), equipped with a 13 mm probe, was used for this purpose. The probe was inserted 1.5 cm inside the samples, and the power and frequency of the device were held at 150 W, and 30 kHz, respectively. The treatments were carried out at 20 °C for 10 min. Subsequently, the samples were centrifuged (4000 rpm/15 min) to remove solids, and filtration (Whatman no. 1) was applied to the supernatant to get clear extracts. All the filtrates were kept at –22 °C until the analyses.

The extraction yields were determined by drying a certain amount of filtrate on a rotary evaporator at 40 °C (IKA KS 400, Staufen, Switzerland). The yields were calculated using the following equation:

(1) $$\mathrm{Extraction}\mathrm{yield}\left(\%\right)=\frac{\mathrm{Weight}\mathrm{of}\mathrm{the}\mathrm{extract}\mathrm{after}\mathrm{evaporation}\;(g)}{\mathrm{Amount}\mathrm{of}\mathrm{dried}\mathrm{sample}\mathrm{used}\;(g)}\times 100$$

### 2.3. Analysis of Total Phenolics

The Folin–Ciocalteau method described by Singleton and Rossi (1965) was used (with slight modification) for the determination of total phenolic contents [21]. Briefly, 25 µL of extract was added to 75 µL of an appropriate solvent (80% acetone, 80% ethanol, or 80% methanol). Then, 0.2 mL of Folin–Ciocalteu reagent and 2 mL of distilled water were added to the mixture and vortexed. After three minutes of incubation, 1 mL of Na_2_CO_3_ (20%, *w/v*) was mixed into the reaction mixture, which was then incubated for one hour at room temperature in the dark. Subsequently, absorbance was monitored at 765 nm with a UV–VIS spectrophotometer (Agilent Model 8453 diode array spectrophotometer, CA, USA). The results were calculated as mg gallic acid equivalent (GAE)/g dry matter, using the standard curve constructed with gallic acid (50–500 ppm) [7].

### 2.4. Analysis of Total Flavonoids

The total flavonoid amounts of the flower and leaf extracts of *P. major* Jacq. were determined using the modified aluminum chloride method [22]. Three hundred µL of distilled water and 30 µL of NaNO_2_ were added to 100 µL of extract, which was then left for 5 min. Then, 30 µL of AlCl_3_, 200 µL of NaOH (1 mM), and 340 µL of distilled water were added, and the absorbance values of the mixtures were measured at 510 nm with a UV–VIS spectrophotometer. The amount of flavonoids was calculated using the quercetin standard curve (10–800 ppm), and the results were represented as mg quercetin (QE)/g dry matter of sample [11].

### 2.5. Analysis of Antioxidant Capacity with the DPPH Method

The 1,1-Diphenyl-2-picrylhydrazyl (DPPH) method was used for the determination of the antioxidant capacity of the extracts according to the Brand–Williams method (1996) with some modifications [23]. The sample (200 µL) was reacted with 3 mL of 0.051 mmol DPPH solution, which was prepared in 70% methanol for 30 min at room temperature in the dark. The control samples were prepared without the extracts. The decrease in absorbance compared to the control sample (created by the scavenging of the radical by the extracts) was measured spectrophotometrically at 517 nm. DPPH radical scavenging activity was calculated in terms of mg trolox equivalent (TE)/g dry matter [24].

### 2.6. UV–VIS Spectral Analysis

All the extracts were properly diluted using 80% acetone, ethanol, or methanol. The solution was then mixed, and absorption spectra were recorded in 200 nm and 600 nm wavelength ranges using a UV–VIS spectrophotometer.

### 2.7. FTIR Analysis

A Fourier transform infrared spectrometer was used to identify the specific peaks characteristic of the functional groups related to the phenolics. The ground powder of the plants’ leaves and flowers, and the extracts obtained from them, were thoroughly examined in the 4000–400 cm^−1^ range using a PerkinElmer Spectrum Two model FTIR spectrometer.

### 2.8. LC–MS Analysis

Bioactive compounds in the ethanol extracts of leaves and flowers *P. major* Jacq. were determined using a Shimadzu LCMS-9030 (Kyoto, Japan) spectrometer equipped with a Q-TOF (Quadrupole-Time of Flight) analyzer. For LC-MS analysis, 200 µL of extract was diluted in 3 mL of 80% ethanol. A CN column (3 µm, 15 cm, 4 mm) was used to separate the components. The mobile phase consisted of acetonitrile and 30 mM formic acid, with a flow rate of 0.5 mL/min and 32 min of analysis time. The column temperature was kept constant at 25 °C, and the sample amount was 10 µL. Linear gradient elution was applied, decreasing the ratio of formic acid as follows: 0–8 min 95%; 8–13 min, 85%; 13–18 min, 70%; 18–20 min, 65%; 20–24 min, 40%; 24–27 min 20%; 27–30 min 10%; and 30–32 min 5% [20,25]. Mass spectra were taken using electrospray ionization (ESI) in both positive and negative ionization modes. The interface voltage was 4.0 kV, and the temperature was 300 °C. The nebulizer gas (helium) flow rate was 3.0 L/min. The resulting peaks were analyzed using Shimadzu LCMS LabSolutions 5.109 software to estimate the existing phenolic compounds. The analyses were performed at the Sakarya University Research Development and Application Center.

### 2.9. Determination of Antibacterial Activity

The antibacterial activity of the extracts was determined using the Kirby–Bauer disc diffusion method [26]. *Bacillus cereus*, *Escherichia coli* O157:H7, *Pseudomonas aeruginosa*, and *Staphylococcus aureus* bacterial strains were used as test organisms to determine the antibacterial activity of *P. major* Jacq. extracts. The methanol extracts of *P. major* Jacq. were dissolved in 3 mL of distilled water and filtered through a cellulose membrane filter (22 µm). The bacterial strains were cultivated in 10 mL of tryptic soy broth at 37 °C for 24 h. Fifty microliters of fresh bacterial cultures were spread onto Mueller Hinton agar and kept for 30 min at room temperature for diffusion. Sterile paper discs (6 mm in diameter) were placed on the agar, and 20 µL of the prepared plant extracts were added to the discs. The Petri dishes were incubated at 37 °C for 24 h [27]. After incubation, the inhibition zones on the agar were evaluated in millimeters. All tests were performed in four replicates.

### 2.10. Statistical Analysis

All analyses were conducted in triplicate, and the results are presented as averages ± standard deviations. The data were statistically analyzed using analysis of variance (ANOVA) with SPSS software (version 11.5, SPSS Inc., Armonk, NY, USA). To identify differences between the samples, Duncan’s multiple range test was applied with a significance level of 0.05 (*p* < 0.05).

## 3. Results and Discussion

### 3.1. Extraction Yield

The extraction yield of polyphenols is influenced by various factors, such as the polarity of the solvent used, extraction procedure, temperature, extraction time, and chemical composition of the sample [28,29]. Thus, the polyphenols from the flowers and leaves of *P. major* Jacq. were extracted using three different solvents with maceration and ultrasonication methods. The yields of the extraction for each condition are given in Table 1. The extraction yields from the flowers were significantly higher than those from the leaves across all tested conditions (*p* < 0.05). The use of methanol with both ultrasonicated and macerated extraction methods resulted in the highest yields, while ethanol used in mac-erated extraction yielded the lowest (*p* < 0.05). It has been reported that carbohydrates and proteins can also be extracted using more polar solvents [29,30,31]. Since methanol has a higher polarity than both ethanol and acetone, the increased yield observed in the methanol extracts may be due to the presence of substances other than polyphenols. Considering the two extraction methods for each solvent, it was found that both methods yielded similar extraction results for leaves and flowers, with no significant differences observed (*p* > 0.05).

### 3.2. Total Phenolic Contents

The total phenolic contents of the samples are shown in Table 2. They ranged between 26.69 and 48.51 mg GAE/g dry matter and the highest TPC was obtained with the ethanol extraction of leaves with both ultrasonication (48.51 ± 1.35 mg GAE/g dry matter) and maceration methods (47.81 ± 1.53 mg GAE/g dry matter) (*p* < 0.05). Regarding the extraction solvents, the efficiency of polyphenol extractions from the leaves in the maceration technique was influenced in the order of ethanol > acetone > methanol, and those from the flowers acetone > ethanol > methanol. In ultrasonicated extraction, the order was ethanol > acetone = methanol for the leaves, and methanol > acetone > ethanol for the flowers (*p* < 0.05). Even though neither method demonstrated significant differences between the TPCs of the leaf extracts of the same solvents (*p* > 0.05), a significantly lower TPC was observed with ultrasonication compared to macerated extraction of the flowers with acetone and ethanol (*p* < 0.05). The phenolic contents of the leaves were significantly higher than those of the flowers with all methods and solvents, showing that the leaves are richer in phenolics. Although certain compounds are found in most parts of a plant, their distribution is restricted to specific locations within the plant anatomy, such as seeds, stems, leaves, bark, etc. [32]. Similarly, Yılmazer Keskin et al. (2024) reported a higher TPC in the leaves of *Spiraea japonica* var. *fortunei* than in the flowers [20]. Mansouri et al. (2021) investigated the extraction of polyphenols from *Hordeum vulgare* L. using various methods, including maceration and ultrasonication using acetone, ethanol, and methanol, and found acetone to be the most efficient solvent for the extraction of polyphenols, followed by ethanol and methanol [33]. Irfan et al. (2022) studied the extraction of polyphenols from *Cymbopogon citratus* leaves with acetone and ethanol using both maceration and ultrasonication methods, and found acetone and ultrasonication to be the most efficient solvent and method, respectively [34]. Contrarily, identical TPC in the leaf extract or lower TPC in the flower with ultrasonication were observed in the current study. The lower TPC in the flower extract with ultrasonication could be due to the existence of higher-bound polyphenols in the flower [35].

The total phenolic contents of various species of *Polygala* demonstrate their significant potential. El Guiche et al. (2015) extracted phenolics from *Polygala balansae* using ultrasound-assisted methanol extraction, revealing a total phenolic content of 14.69 µg GAE/mg of dry matter [36]. In another study, the methanol extract of *Polygala tenuifolia* Willd. exhibited a total phenolic content of 0.82 g/100 g of dry matter [37]. Additionally, Li et al. (2018) determined that the root of *Polygala tenuifolia* Willd. contained a total phenolic content of 49.96 mg GAE/g of dry matter [38]. Furthermore, the ethanolic extract of *Polygala chinensis* demonstrated a total phenolic content of 88.2 mg GAE/g of extract [39].

### 3.3. Total Flavonoid Contents

As in TPC, the flavonoid contents of the leaves were significantly higher than those of the flowers (*p* < 0.05) (Table 3). The highest flavonoid contents were detected when acetone was used with both extraction methods, and the lowest flavonoid contents were obtained with methanol. This can be explained by the fact that flavonoids are more soluble in acetone, which is less polar than methanol and ethanol. When the effects of maceration and ultrasonication methods were compared, it was seen that there were no significant differences in the acetone, ethanol, and methanol extracts of flowers with both methods or in the methanol extracts of the leaves (*p* > 0.05). However, significant differences were observed with the other samples (*p* < 0.05). Similarly, Iloki-Assanga (2015) studied the flavonoids of *Bucida buceras* L. leaves using acetone, ethanol, and methanol as extraction solvents and found acetone to be the most effective extraction solvent, followed by ethanol and methanol [32]. Anokwuru et al. (2011) investigated the TFCs of three Nigerian medicinal plants (*Acalypha wilkesiana* leaf, *Azadirachta indica* stem, and *Solanum scrabrum* leaf) using acetone, ethyl acetate, ethanol, and methanol as solvents [40]. The authors reported obtaining the highest TFC with methanol for *A. wilkesiana*, ethyl acetate for *S. scabrum*, and ethanol for *A. indica*. They also reported a negative correlation between the TPC and TFC of *S. scabrum*. The results indicated that the choice of solvent for the extraction is also related to the plant material.

In their study, Brighente et al. (2007) analyzed the flavonoid contents of the aerial parts of two *Polygala* species, *Polygala cyparissias* and *Polygala sabulosa* [41]. They used a 75% ethanol extraction method, which yielded flavonoid contents of 12.08 mg QE per dry extract for *P. cyparissias* and 24.68 mg QE per dry extract for *P. sabulosa*. Additionally, the ethanolic extract of *Polygala chinensis* was reported to have a flavonoid content of 36.7 mg QE per 100 g of dry extract [39]. Furthermore, the flavonoid content of the ultrasound-assisted methanol extract of *Polygala balansae* was found to be 29.20 µg QE per mg of dry matter, which is consistent with our findings [36].

### 3.4. Antioxidant Activity

The antioxidant activities of the samples, as measured with the DPPH method in terms of trolox equivalent (Table 4), ranged between 0.65 ± 0.02 and 0.86 ± 0.0 mg TE/g dry matter. Overall, there were no huge activity variations between the samples. It must be noted that, unlike the phenolic and flavonoid contents, the DPPH radical scavenging activities of the flower extracts were higher than those of the leaf extracts, except for the ultrasonicated methanol extract of leaves (*p* < 0.05). This can be explained by the fact that antioxidants found in the flowers are more soluble in the solvents used in the study. Phytochemicals, particularly phenolic compounds and their derivatives, are responsible for exhibiting antioxidant activity due to their redox properties. The hydroxyl groups present in these compounds enhance their ability to scavenge free radicals [42].

### 3.5. Antimicrobial Activity

The use of natural antimicrobial agents to combat pathogenic microorganisms and replace antimicrobial chemicals, as well as antibiotics, has been a subject of great attention [43]. Medicinal plants have been shown to have fewer side effects than antibiotics [44]. In the current study, the antimicrobial activities of the methanol extracts of the leaf and flowers of *P. major* Jacq. were tested on two Gram-positive (*B. cereus* and *S. aureus*), and two Gram-negative (*P. aeruginosa* and *E. coli* O157:H7) bacteria (Table 5). The antimicrobial activity of the leaf methanol extract was detected against *B. cereus*, *S. aureus*, and *P. aeruginosa*. However, it did not inhibit the growth of *Escherichia coli* O157:H7. On the other hand, flower methanol extracts did not exhibit any antimicrobial activity against the bacteria tested. This can be attributed to the fact that many of the phytochemicals with apparent antimicrobial effects are concentrated in the leaf [45]. It has been suggested that some phytochemicals, such as terpenoids, alkaloids, and phenolic compounds, can disrupt the microbial cell membrane by interacting with enzymes and proteins in the membrane. This leads to a flux of protons toward the cell exterior, inducing cell death or inhibiting the enzymes necessary for amino acid biosynthesis [46]. Similarly, methanol extracts from the leaves of red *Clerodendrum paniculatum*, *Arytera littoralis*, and *Bauhinia kockiana* demonstrated antibacterial activity against *Bacillus subtilis*. In contrast, the flower extracts of these plants showed no antibacterial effects. Additionally, neither the leaf nor the flower extracts were effective in inhibiting *Escherichia coli* [47].

### 3.6. UV–VIS Spectra

**Figure 1:**
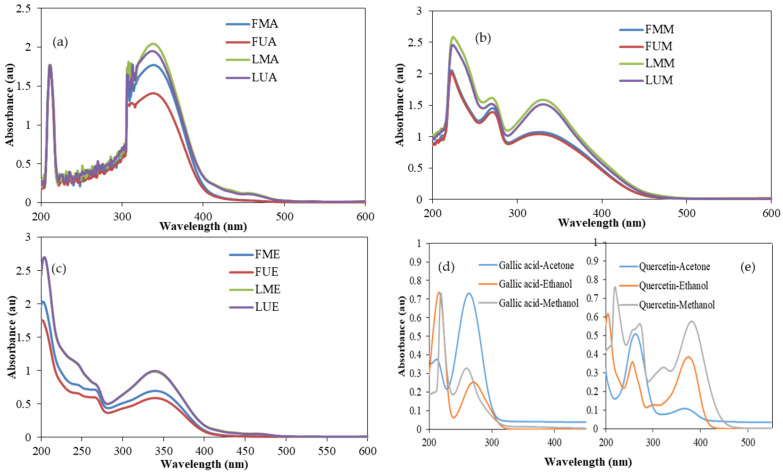
UV–VIS spectra of the flower and leaf extracts of *Polygala major* Jacq. using different methods and solvents. (**a**) acetone extracts; (**b**) ethanol extracts; (**c**) methanol extracts; (**d**) gallic acid standard; (**e**) quercetin standard. (FMA: Flower macerated with acetone; FUA: Flower ultrasonicated with acetone; LMA: Leaf macerated with acetone; LUA: Leaf ultrasonicated with acetone; FME: Flower macerated with ethanol; FUE: Flower ultrasonicated with ethanol; LME: Leaf macerated with ethanol; LUE: Leaf ultrasonicated with ethanol; FMM: Flower macerated with methanol; FUM: Flower ultrasonicated with methanol; LMM: Leaf macerated with methanol; LUM; Leaf ultrasonicated with methanol).

UV–VIS spectra of *P. major* Jacq. flower and leaf extracts of acetone, ethanol, and methanol using maceration and ultrasonication are shown in Figure 1. Spectra of gallic acid and quercetin standards in the same solvents are also given in the figure. Wide absorbance bands were observed between 300 and 385 nm in all the samples tested, which indicated the presence of flavonoids. Flavonoids exhibit two typical peaks in their UV spectra: flavones are found in the 310–350 nm range in Band A, while flavonols are found in the 350–385 nm range. A similar absorption band was detected in the quercetin standard. Other phenolics (Band B) absorb UV light in the 250–290 nm range [48,49]. The methanol and ethanol extracts exhibited absorption bands at those wavelengths, while acetone extracts did not. However, acetone extracts had absorption peaks at around 310–320 nm. The standards used to determine phenolic-flavonoid compounds are gallic acid and quercetin. These standards can be dissolved in methanol, ethanol, and acetone, and their UV spectra can be seen in Figure 1. Gallic acid exhibits two peak points at 216–219 nm and 260–271 nm, while quercetin exhibits four peaks at approximately 204–219 nm, 256–272 nm, 305–322 nm, and 374–382 nm.

### 3.7. FTIR Analysis

**Figure 2:**
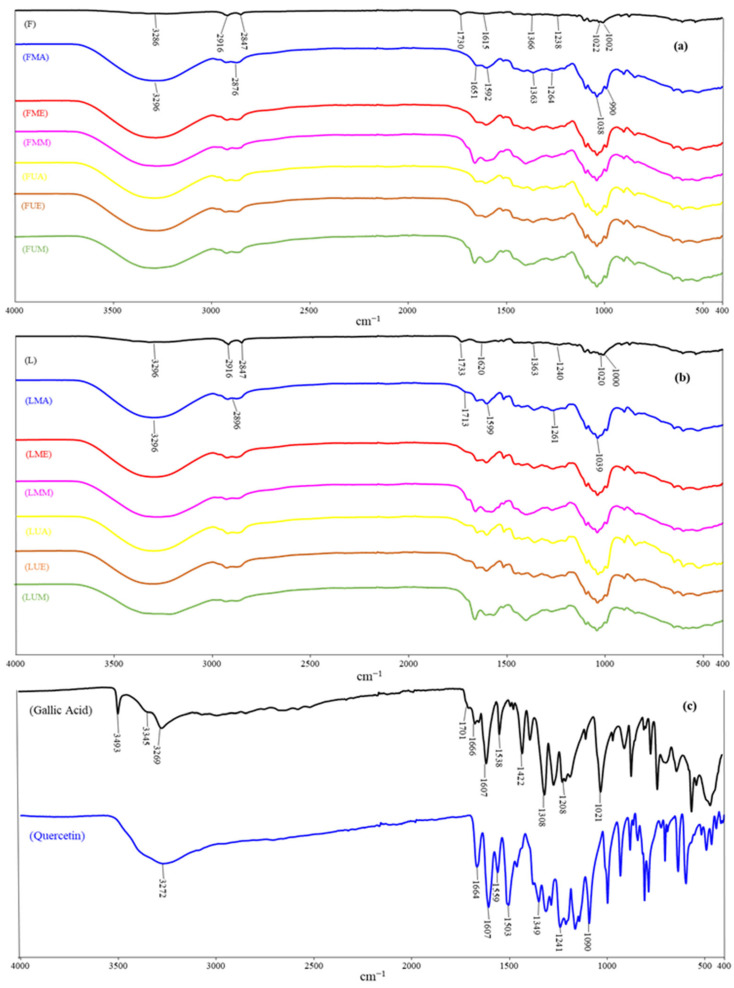
FTIR spectra of the flower and leaf extracts of *Polygala major* Jacq. using different methods and solvents. (**a**) raw flower, macerated flower extracts, and ultrasonicated flower extracts; (**b**) raw leaf, macerated leaf extracts, and ultrasonicated leaf extracts; (**c**) gallic acid and quercetin standards. (FMA: Flower macerated with acetone; FUA: Flower ultrasonicated with acetone; LMA: Leaf macerated with acetone; LUA: Leaf ultrasonicated with acetone; FME: Flower macerated with ethanol; FUE: Flower ultrasonicated with ethanol; LME: Leaf macerated with ethanol; LUE: Leaf ultrasonicated with ethanol; FMM: Flower macerated with methanol; FUM: Flower ultrasonicated with methanol; LMM: Leaf macerated with methanol; LUM; Leaf ultrasonicated with methanol).

FTIR spectra of the acetone, ethanol, and methanol extracts of the leaves and flowers of *P. major* Jacq. are presented in Figure 2, together with those of the raw leaf and flower powders and the standards (gallic acid and quercetin). Phenolic compounds and -OH groups in alcohols exhibit a broad O-H stretching vibration between 3300–3200 cm^−1^ in all samples [50]. The 2916 and 2847 cm^−1^ peaks in the raw flower and leaf samples represent the stretching vibration of the non-aromatic C-H groups. These peak intensities are lower in flower and leaf extracts. The peaks at 1733, 1713, 1707, and 1697 cm^−1^ in the raw leaf, acetone, ethanol, and methanol extracts, respectively, represent the stretching vibration of the carbonyl group (C=O). The stretching vibration of the aromatic C=C group appears at 1620, 1599, 1602, and 1580 cm^−1^ in the raw leaf, acetone, ethanol, and methanol extracts, respectively. The 1500–500 cm^−1^ region represents the fingerprint region specific to a particular compound. While almost similar spectra are obtained from leaf extracts using acetone, ethanol, and methanol in this region, the raw leaf sample is slightly different. The peaks at 1020 and 1240 cm^−1^ represent the stretching vibration of the C-O group, while the peaks around 1300 cm^−1^ represent the bending vibration of the O-H group. The peaks at around 1200 cm^−1^ and 1000 cm^−1^ belong to the stretching vibration of the C-O-C group [51].

The FTIR spectra of the flower and leaf samples of the raw plant, as well as its acetone, ethanol, and methanol extracts, show the presence of the same functional groups in both the leaves and flowers. However, the intensity of the O-H stretching vibration (3300–3200 cm^−1^) is higher in the raw leaves than in the raw flowers. Conversely, the peaks at 2917 and 2849 cm^−1^ in the raw flowers are more intense than the same peaks in the raw leaves. Figure 2a,b show that the FTIR spectra of the ultrasonic extracts are very similar to those obtained by conventional extraction.

The FTIR spectra of gallic acid and quercetin are shown in Figure 2c. In the gallic acid spectrum, the peaks at 3493, 3345, and 3269 cm^−1^ correspond to different O-H groups in the structure of gallic acid, while the peak at 1701 cm^−1^ corresponds to the C=O group and the peak at 1615 cm^−1^ corresponds to the C=C bond in alkenes [52]. The peaks at 1607 and 1538 cm^−1^ correspond to the stretching vibrations of the C-C bonds in the aromatic ring of gallic acid, while the peaks in the 1300–1000 cm^−1^ region correspond to the stretching vibrations of the C-O group and the bending vibrations of the O-H bonds [53]. In the FTIR spectrum of quercetin, the stretching and bending vibrations of the O-H group appear at 3272 cm^−1^ and 1349 cm^−1^, respectively. The peak at 1664 cm^−1^ corresponds to the C=O group, while the peaks at 1607 cm^−1^ and 1559 cm^−1^ correspond to the vibration of the aromatic C=C and C-C groups, respectively. The peaks at 1241 cm^−1^ and 1090 cm^−1^ correspond to the vibration of the C-O group. The main peaks obtained using FTIR spectra of flower, leaf, and whole extracts of *P. major* Jacq. are summarized in Table 6.

### 3.8. LC–MS Analysis of Polyphenols

**Figure 3:**
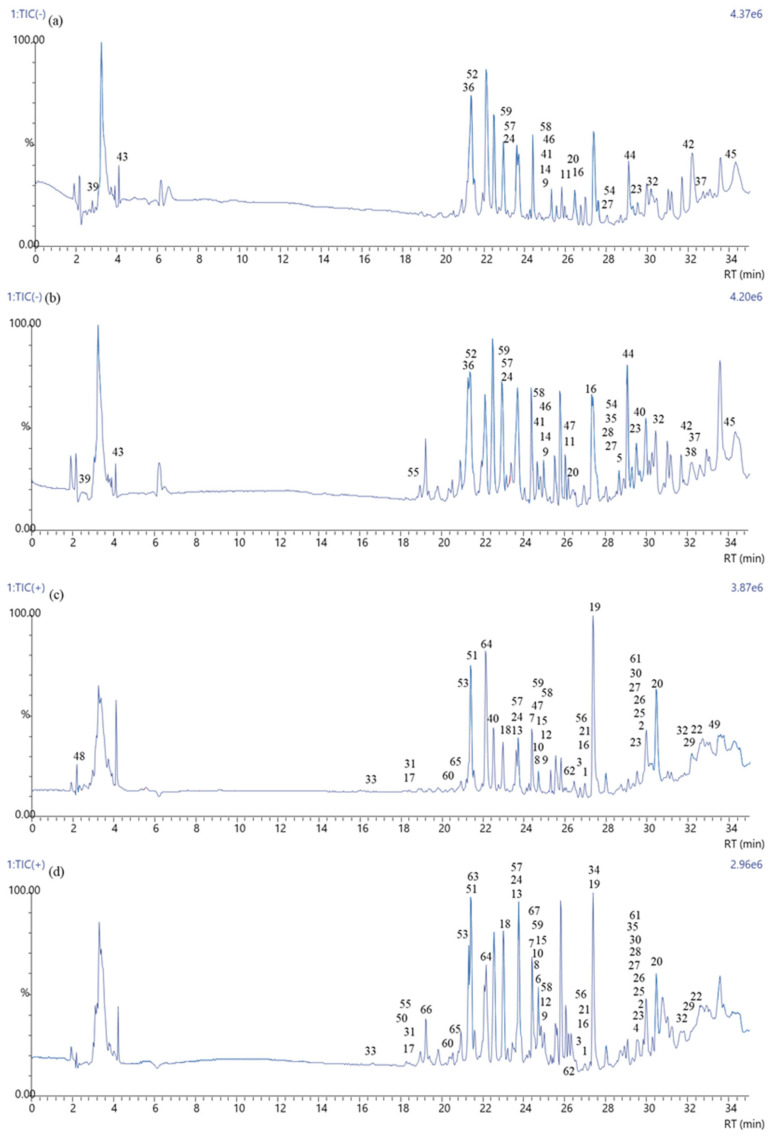
LC–MS chromatograms of *Polygala major* Jacq. extracts. (**a**) negative ion chromatogram of the flower extract, (**b**) negative ion chromatogram of the leaf extract, (**c**) positive ion chromatogram of the flower extract, (**d**) positive ion chromatogram of the leaf extract.

The LC–MS negative and positive ion chromatograms of ethanol extracts of *P. major* Jacq. leaves and flowers are shown in Figure 3. A total of 55 and 65 phenolic compounds were detected in the flowers and leaves of *P. major* Jacq., respectively (Table 7). Molecules 4–6, 28, 34–35, 38, 50, 55, 63, 66, and 67 were found only in the leaf, while 48 and 49 were present only in the flower. The rest of the molecules were found in both the leaf and flower extracts. The molecular peaks of 5, 9, 11, 14, 36–39, 41–46, 52, and 54 were observed only in negative ion mode. Positive ion mode was also investigated due to possible non-ionized molecules. Molecules 1–8, 10, 12–13, 15, 17–19, 21–22, 25–26, 29–31, 33–34, 48–51, 53, 56, and 60–67 were identified only in positive ion mode. The remaining 15 molecules were observed in both positive and negative ion modes. The chemical structures of the molecules that originated from the chromatograms are given in Supplementary Figure S1. Although more phytochemicals were found in the leaf extract, important pharmaceutical compounds were determined in both extracts. For example, eupatorin (mol # 1), which has been detected in both flower and leaf, is a flavonoid with a wide range of phytomedicine activities and has been reported to have significant contributions in the treatment of prostate cancer [54]. Sinensetin (2) is known to have strong anticancer activities and several other pharmacological benefits, and it plays an essential role in targeted activities with minimal toxicity [55]. Acacetin (7), as reported in a study by Singh et al. (2020), has powerful anti-inflammatory and anticancer activity [56]. Another study reported that ombuin (11) has a broad-spectrum antibacterial effect [57]. Vanillic acid (47) has been reported to have anticancer, anti-obesity, antidiabetic, antibacterial, anti-inflammatory, and antioxidant effects, and is a flavoring agent in various food products [58]. Sinapic (40) and ferulic acid (32) phenethyl esters have been reported to improve cholesterol and steroid biosynthesis in testicular Leydig cells [59]. In a study by Rahaman et al. (2023), it was reported that gallic acid (29) acted as an adjuvant with vancomycin to overcome antibiotic resistance and that this drug could be administered as an alternative treatment for staphylococcal joint infections by intra-articular injection [60]. Daidzein (27) has been shown to reduce cell death caused by nitric oxide (NO) stress and even increase mitochondrial content in the presence of NO [61]. Naturally occurring and synthetic chalcone derivatives exhibit biological activities against cancer properties, such as proliferation, angiogenesis, metastasis, inflammation, and cancer epigenetics regulation [62]. Subscandenin (13) has been shown to have antioxidant and antidiabetic activity and inhibit free radical scavenging and α-glucosidase enzyme [63]. Saquiba et al. (2020) reported that persicogenin (14) has anticancer effects in MCF-7, HeLa, and HT-29 cells and can be applied as a bioactive therapeutic agent [64]. Apigenin (3) has been shown to reverse hypoxia-induced resistance in cancer cells [65]. Naringenin (found only in leaf extract) (4) and quercetin (6) are phenolic compounds known to improve glucose-stimulated insulin secretion and glucose sensitivity in INS-1E cells [66]. Another study determined that 5,7-dihydroxy-3,4′-dimethoxyflavone (9) has a high antimycobacterial effect [67]. Luteolin (5, 7, 3′,4′-tetrahydroxy flavone) (18) has become of interest within the biological sciences owing to its antioxidant and antiallergic properties [68].

## 4. Conclusions

Polyphenols from the leaves and flowers of *P. major* Jacq. were extracted successfully using three different solvents (acetone, ethanol, and methanol) and by applying two extraction methods (maceration and ultrasonication) for the first time in this study. The leaf of the plant had higher polyphenols than the flower. There were no significant differences between the maceration and ultrasonication methods. Even though there were no big variations in total phenolic contents between the samples using different solvents, the flavonoid content in the acetone extracts was significantly increased compared to ethanol and methanol. All the extracts had antioxidant activities, and the leaf extracts had antibacterial activities against *B. cereus*, *S. aureus*, and *P. aeruginosa*. The presence of polyphenols was also confirmed with UV–VIS spectra and FTIR analysis. Various phenolic substances, including eupatorin, sinensetin, acacetin, ombuin, vanillic acid, sinapic acid, ferulic acid, daidzein, subscandenin, persicogenin, apigenin, naringenin, and luteolin, have been identified both in the flower and leaf. These findings highlight the importance of *P. major* Jacq. in phytochemical research and its potential applications in related fields.

## Figures and Tables

**Table 1:** Macerated and ultrasonicated extraction efficiency of *P. major* Jacq. using acetone, ethanol, and methanol.

Solvent, 80%	Sample	Extraction Efficiency (%)	
		Macerated Extraction	Ultrasonic Extraction
Acetone	Flower	36.72 ^bA^* ± 1.82	36.80 ^bA^* ± 0.84
	Leaf	27.52 ^cB^ ± 2.36	30.20 ^cB^ ± 1.13
Ethanol	Flower	28.64 ^cA^ ± 2.12	28.80 ^cA^ ± 0.82
	Leaf	21.72 ^dB^* ± 1.78	22.90 ^dB^* ± 0.94
Methanol	Flower	42.44 ^aA^* ± 2.44	44.02 ^aA^* ± 0.83
	Leaf	28.44 ^cB^ ± 2.48	31.38 ^cB^ ± 1.06

Lowercase letters within the same column represent the significant differences between different solvents in each extraction method (macerated and ultrasonicated) while the uppercase letters in the same solvent indicate the differences between the two extraction methods for each solvent (*p* < 0.05). Asterisks (*) show the significant differences between the flowers and leaves (*p* < 0.05). The data presented are the means and ±standard deviation of three samples (n = 3).

**Table 2:** Total phenolic contents of flowers and leaves of *Polygala major* Jacq. extracted using different solvents (acetone, ethanol, and methanol) and methods (maceration and ultrasonication).

Solvent, 80%	Sample	Total Phenolic Content (mg GAE/g Dry Matter)	
		Macerated Extraction	Ultrasonic Extraction
Acetone	Flower	37.81 ^cB^* ± 2.00	29.04 ^cdC^* ± 2.93
	Leaf	45.38 ^abA^* ± 2.67	42.18 ^bA^* ± 1.29
Ethanol	Flower	35.71 ^cdB^* ± 0.90	26.69 ^dC^* ± 1.35
	Leaf	47.81 ^aA^* ± 1.53	48.51 ^aA^* ± 1.35
Methanol	Flower	32.99 ^dB^* ± 1.65	31.57 ^cB^* ± 0.96
	Leaf	43.30 ^bA^* ± 1.42	44.27 ^bA^* ± 2.45

Lowercase letters within the same column represent the significant differences between different solvents in each extraction method (macerated and ultrasonicated) while the uppercase letters in the same solvent indicate the differences between the two extraction methods for each solvent (*p* < 0.05). Asterisks (*) show the significant differences between the flowers and leaves (*p* < 0.05). The data presented are the means and ±standard deviation of three samples (n = 3).

**Table 3:** Total flavonoid contents of flowers and leaves of *Polygala major* Jacq. extracted using different solvents (acetone, ethanol, and methanol) and methods (maceration and ultrasonication).

Solvent, 80%	Sample	Total Flavonoid Content (mg QE/g Dry Matter)	
		Macerated Extraction	Ultrasonic Extraction
Acetone	Flower	5.14 ^dC^* ± 0.13	5.48 ^dC^* ± 0.22
	Leaf	16.56 ^aA^* ± 0.72	15.14 ^aB^* ± 0.95
Ethanol	Flower	4.47 ^dC^* ± 0.08	4.27 ^eC^* ± 0.18
	Leaf	10.97 ^bA^* ± 0.52	9.95 ^bB^* ± 0.24
Methanol	Flower	4.17 ^dB^* ± 0.39	5.02 ^deB^* ± 0.40
	Leaf	7.93 ^cA^* ± 0.61	7.48 ^cA^* ± 0.22

Lowercase letters within the same column represent the significant differences between different solvents in each extraction method (macerated and ultrasonicated) while the uppercase letters in the same solvent indicate the differences between the two extraction methods for each solvent (*p* < 0.05). Asterisks (*) show the significant differences between the flowers and leaves (*p* < 0.05). The data presented are the means and ±standard deviation of three samples (n = 3).

**Table 4:** DPPH radical scavenging activities of flowers and leaves of *Polygala major* Jacq. extracted using different solvents (acetone, ethanol, and methanol) and methods (maceration and ultrasonication).

Solvent, 80%	Sample	DPPH Scavenging Activity (mgTE/g Dry Matter)	
		Macerated Extraction	Ultrasonic Extraction
Acetone	Flower	0.74 ^bB^* ± 0.00	0.74 ^bA^* ± 0.00
	Leaf	0.66 ^dC^* ± 0.01	0.65 ^dC^* ± 0.02
Ethanol	Flower	0.72 ^bB^* ± 0.01	0.74 ^bA^* ± 0.00
	Leaf	0.69 ^cC^* ± 0.01	0.70 ^cC^* ± 0.01
Methanol	Flower	0.80 ^aB^* ± 0.00	0.77 ^bC^* ± 0.00
	Leaf	0.81 ^bD^* ± 0.01	0.86 ^aA^* ± 0.00

Lowercase letters within the same column represent the significant differences between different solvents in each extraction method (macerated and ultrasonicated) while the uppercase letters in the same solvent indicate the differences between the two extraction methods for each solvent (*p* < 0.05). Asterisks (*) show the significant differences between the flowers and leaves (*p* < 0.05). The data presented are the means and ±standard deviation of three samples (n = 3).

**Table 5:** Antimicrobial activity of the methanol extracts of *Polygala major* Jacq. on selected pathogenic bacteria.

Bacteria	Zone of Inhibition (mm)	
	Leaves	Flowers
*Bacillus cereus*	10.00 ± 0.0	-
*Staphylococcus aureus*	9.75 ± 0.5	-
*Pseudomonas aeruginosa*	10.50 ± 0.6	-
*Escherichia coli* O157:H7	-	-

**Table 6:** The main peaks deduced from the FTIR spectra of the flower, leaf, and all extracts of *Polygala major* Jacq.

*P. major* Jacq.		O-H Stretching (cm^−1^)	C-H Stretching (cm^−1^)	C=O Stretching (cm^−1^)	C=C Stretching (cm^−1^)	C-O Stretching (cm^−1^)	O-H Bending (cm^−1^)	C-O-C Stretching (cm^−1^)
	Flowers	3296	2916, 2817	1760	1615	1238, 1022	1366	1002
	Leaves	3296	2916, 2817	1733	1620	1240, 1020	1363	1000
Macerated Extraction	Flowers–acetone	3296	2876	1651	1592	1264, 1038	1363	990
	Flowers–ethanol	3296	2876	1661	1592	1264, 1038	1363	990
	Flowers–methanol	3296	2876	1697	1592	1264, 1038	1390	990
	Leaves–acetone	3296	2896	1713	1599	1261, 1039	1366	990
	Leaves–ethanol	3296	2896	1707	1602	1261, 1039	1366	990
	Leaves–methanol	3296	2896	1697	1580	1261, 1039	1388	990
Ultrasonic Extraction	Flowers–acetone	3285	2896	1648	1599	1265, 1036	1367	990
	Flowers–ethanol	3285	2896	1648	1599	1265, 1036	1367	990
	Flowers–methanol	3285	2896	1661	1599	1265, 1036	1386	990
	Leaves–acetone	3289	2892	1648	1599	1265, 1036	1361	987
	Leaves–ethanol	3289	2892	1648	1599	1265, 1036	1361	987
	Leaves–methanol	3289	2892	1661	1599	1265, 1036	1395	987

**Table 7:** Profile of polyphenols detected in the ethanol extract of *Polygala major* Jacq. by LC–MS.

No	Compound	MW, (amu)	Flower and /or Leaf	RT * (min)	(M^+^)	(M+H)^+^	(M-H)^−^
1	Eupatorin (3′,5-dihydroxy-4′,6,7-trimethoxyflavone)	344.30	F-L	27.39	344	-	-
2	Sinensetin (3′,4′,5,6,7- pentamethoxyflavone)	372.36	F-L	29.96	372	-	-
3	Apigenin (5,7,4′- trihydroxyflavone)	270.24	F-L	26.97	-	271	-
4	Naringenin (4′,5,7-trihydroxyflavanone)	272.25	L	28.90	-	273	-
5	3,5,7-Trihydroxy-2-phenylchroman-4-one	272.25	L	28.87	-	-	271
6	Quersetin (3,5,7,3′,4′-pentahydroxyflavone)	302.24	L	24.83	-	303	-
7	Acacetin (5,7-dihydroxy-4′-methoxyflavone)	284.26	F-L	24.27	-	285	-
8	3,5,7-Trihydroxy-4′-methoxyflavone	300.26	F-L	24.46	-	301	-
9	5,7-Dihydroxy-3′,4′-dimethoxyflavone	314.29	F-L	25.54	-	315	-
9	5,7-Dihydroxy-3′,4′-dimethoxyflavone	314.29	F-L	25.51	-	-	313
10	5-Hydroxy-6,7,4′-trimethoxyflavanone	330.33	F-L	24.46	-	331	-
11	Ombuin(3,3′,5-trihydroxy-7,4′-dimethoxyflavone)	330.29	F-L	26.39	-	-	329
12	6-Methoxyluteolin	316.26	F-L	25.54	316	-	-
13	Subscandenin(5,7-dihydroxy-8,4′-dimethoxyflavanone)	316.31	F-L	23.76	-	317	-
14	Persikogenin(5,3′-dihydroxy-7,4′-dimethoxyflavanone)	316.31	F-L	25.51	-	-	315
15	Isoquercitrin (quercetin-3-O-glucopyranoside)	464.38	F-L	24.83	-	465	-
16	Odoratin(2-hydroxy-4′,5′,6,4-tetramethoxy chalcone)	344.36	F-L	27.39	344	-	-
16	Odoratin(2-hydroxy-4′,5′,6,4-tetramethoxy chalcone)	344.36	F-L	27.37	-	-	343
17	3,5-Dihydroxy-7-methoxy-flavanone	286.28	F-L	18.41	-	287	-
18	Luteolin (5, 7, 3′,4′-tetrahydroxy flavone)	286.24	F-L	23.53	-	287	-
19	4′,5,6,7-tetramethoxyflavone(scutellarein tetramethyl ether)	342.34	F-L	27.82	-	343	-
20	2′,4′,6′,3,4-Pentahydroxychalcone)	288.25	F-L	30.45	288	-	-
20	2′,4′,6′,3,4-Pentahydroxychalcone	288.25	F-L	26.93	-	-	287
21	Quercetin-7,3′,4′-trimethylether	344.32	F-L	27.39	344	-	-
22	Mirisetin	318.24	F-L	32.62	318	-	-
23	Chalcone	208.26	F-L	29.57	-	209	-
23	Chalcone	208.26	F-L	29.50	-	-	207
24	Quercetin 3,7-diglucoside	626.52	F-L	23.76	626	-	-
24	Quercetin 3,7-diglucoside	626.52	F-L	23.69	-	-	625
25	Luteolin-7-O-glucuronide	462.36	F-L	28.90	-	463	-
26	Quercetin 3-O-β-D-glucopyranoside	464.38	F-L	28.90	464	-	-
27	Daidzein (7,4′-dihydroxyisoflavone)	254.24	F-L	28.69	-	255	-
27	Daidzein (7,4′-dihydroxyisoflavone)	254.24	F-L	28.64	-	-	253
28	(+)-Catechin	290.27	L	28.69	-	291	-
28	(+)-Catechin	290.27	L	28.50	-	-	289
29	Gallic acid	170.12	F-L	32.25	170	-	-
30	Syringic acid	198.17	F-L	28.69	-	199	-
31	Protocatechuic acid	154.22	F-L	18.41	154	-	-
32	Ferulic acid	194.18	F-L	31.81	194	-	-
32	Ferulic acid	194.18	F-L	30.42	-	-	193
33	Veratric acid	182.17	F-L	16.62	182	-	-
34	Benzenepropanoic acid	150.17	L	27.82	-	151	-
35	5-Hydroxyferulic acid	210.18	L	29.52	210	-	-
35	5-Hydroxyferulic acid	210.18	L	28.64	-	-	209
36	Eriocitrin (Eriodictyol 7-O-rutinoside)	596.17	F-L	21.25	-	-	595
37	Apigenin-7,4′-dimethyl ether	298.29	F-L	33.04	-	-	297
38	4-Methoxycinnamic acid	178.18	L	32.60	-	-	177
39	Caffeic acid	180.16	F-L	3.05	-	-	179
40	Sinapic acid	224.21	L	29.67	-	-	223
40	Sinapic acid	224.21	F	22.498	-	225	-
41	Myricetin 3-β-D-glucopyranoside	480.38	F-L	25.27	-	-	479
42	Kaempferol-4′-glucoside	448.38	F-L	32.18	-	-	447
43	*p*-Coumaric acid	164.16	F-L	4.10	-	-	163
44	*p*-Hydroxybenzoic acid	138.12	F-L	29.27	-	-	137
45	3-phenyl-1-(2,4,6-trihydroxyphenyl)prop-2-en-1-one	256.25	F-L	34.31	-	-	255
46	AgestricinC(6-hydroxy-5,7,3′,4′-tetramethoxyflavanone)	360.36	F-L	25.51	-	-	359
47	Vanilic acid	168.18	L	26.39	-	-	167
47	Vanillic acid	168.18	F	25.390	-	169	-
48	5-hydroxy-6,7,3′,4′-tetramethoxyflavone	358.30	F	2.175	358	-	-
49	Cinnamic acid	148.16	F	33.713	-	149	-
50	Nevadensin(5,7-dihydroxy-6,8,4′-trimethoxyflavone)	344.32	L	18.26	344	-	-
51	Hesperitin (hesperetin-7-rutinoside)	610.60	F-L	21.41	-	611	-
52	Quercetin-3-O-α-L-rhamnopyranosyl-(1–6)-β-D-galactopyranoside	610.52	F-L	21.40	-	-	609
53	Vicenin-2 (apigenin-6,8-di-C-glucoside)	594.50	F-L	22.15	-	595	-
54	Padmatin (taxifolin-7-methyl ether)	318.28	F-L	28.69	-	-	319
55	Apigenin-5-O-β-D-glucopyranoside	432.40	L	18.96	-	433	-
55	Apigenin-5-O-β-D-glucopyranoside	432.40	L	18.30	-	-	431
56	Quercetin-3-O-β-D-glucuronide	478.36	F-L	26.97	-	479	-
57	Narcissin (isorhamnetin-3-rutinoside)	624.54	F-L	23.76	-	625	-
57	Narcissin (isorhamnetin-3-rutinoside)	624.54	F-L	23.69	-	-	623
58	Quercetin 3-O-α-L-arabinoside	434.35	F-L	25.30	-	435	-
58	Quercetin 3-O-α-L-arabinoside	434.35	F-L	25.27	-	-	433
59	Quercetin-3-O-rutinoside-7-O-glycoside	772.66	F-L	24.71	772	-	-
59	Quercetin-3-O-rutinoside-7-O-glycoside	772.66	F-L	23.38	-	-	771
60	Spireasalicin (Kuersetin-3-O-[6″-(4′′′-hidroksi-2′′′-metilenbutirol)]- β-D-glukopiranosid)	562.48	F-L	20.42	-	563	-
61	(+)-Catechin-7-α-L-arabinofuranoside	422.38	F-L	28.90	-	423	-
62	3,5-Dicaffeoylquinic acid	516.45	F-L	26.53	516	-	-
63	Cichoric acid	474.37	L	21.60	-	475	-
64	5-O-feruloylquinic acid	368.30	F-L	22.55	-	369	-
65	Apigenin-7-O-glucuronide	446.36	F-L	20.93	446	-	-
66	Amentoflavone	538.45	L	19.22	538	-	-
67	Hiperosit (Quercetin-3-galactoside)	464.38	L	24.83	-	465	-

* Retention Time.

## Data Availability

The original contributions presented in this study are included in the article/Supplementary Materials. Further inquiries can be directed to the corresponding author.

## Supplementary Materials

The following supporting information can be downloaded at: https://www.mdpi.com/article/10.3390/antiox14020153/s1, Figure S1: LC–MS spectra of all components.
