# Supplementary material for: Analysis of Polyphenols from Polygala major Jacq
Source: Antioxidants (Basel). 2025 Jan 27;14(2):153. doi: 10.3390/antiox14020153 (PMC11851501; doi:10.3390/antiox14020153)

Figure S1. LC–MS spectra of all components.

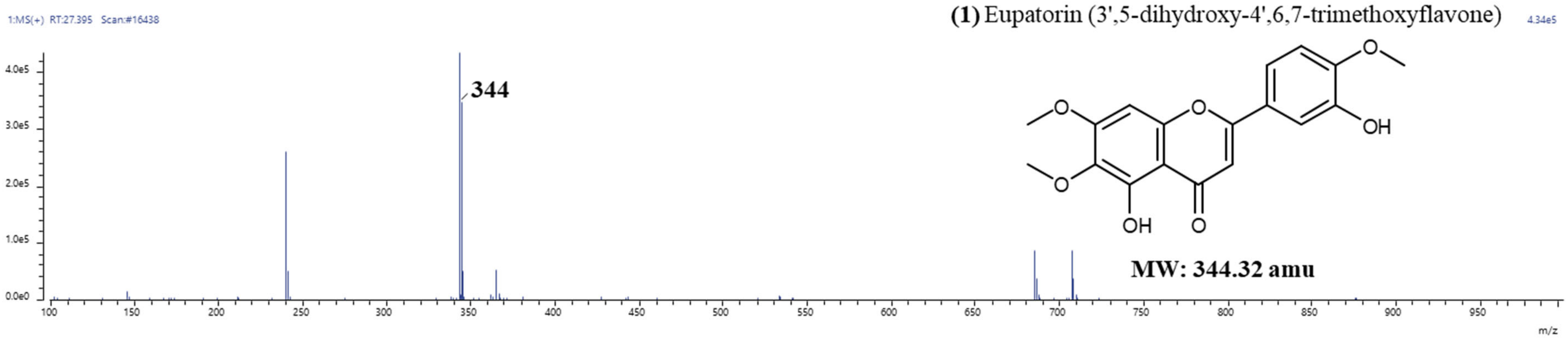

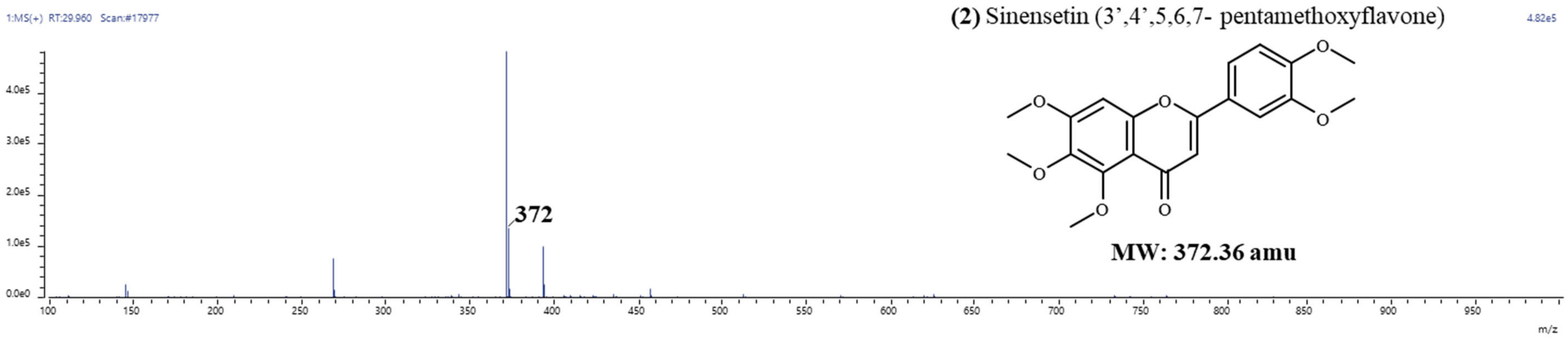

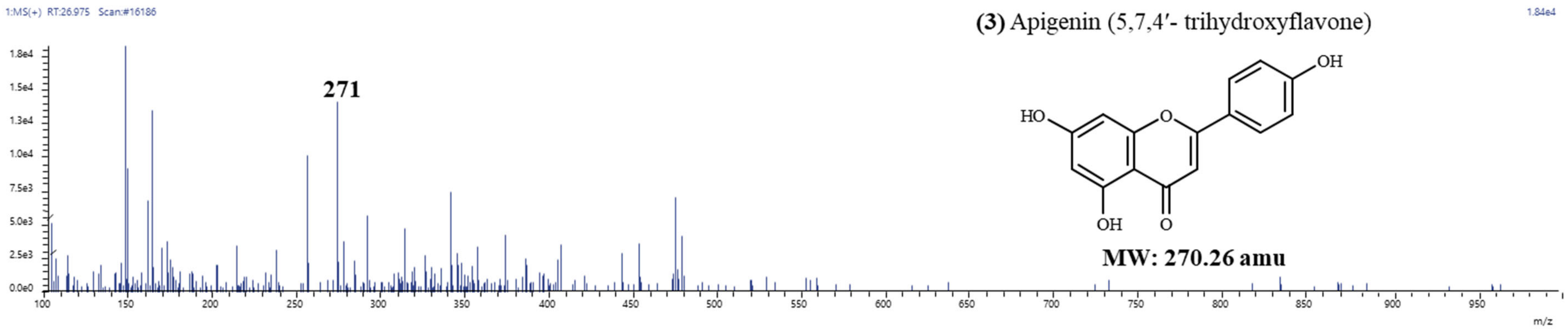
1:MS(+) RT:26.975 Scan:#16186
(3) Apigenin (5,7,4′- trihydroxyflavone)
1.84e4
271
HO
OH
O
OH
O
MW: 270.26 amu
m/z

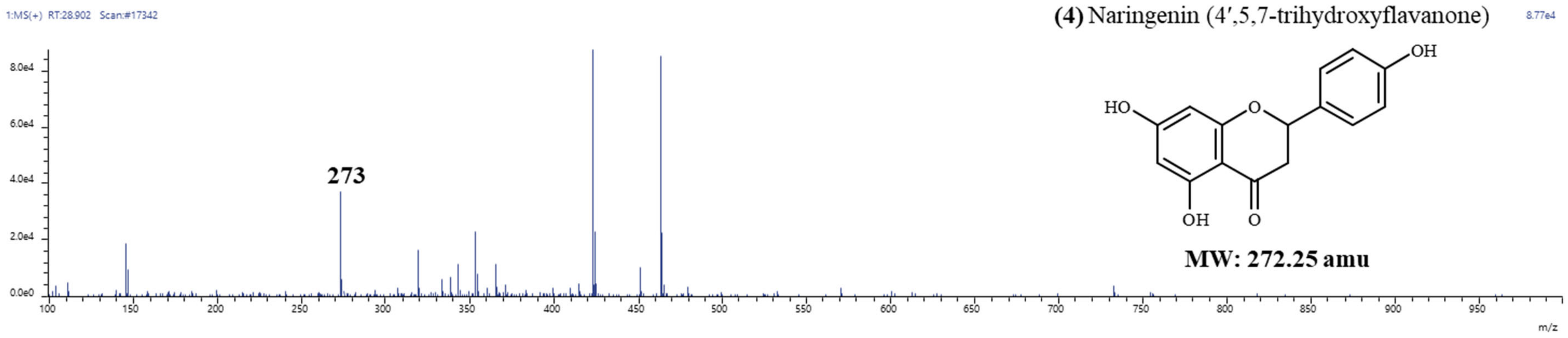
1:MS(+) RT:28.902 Scan:#17342
(4) Naringenin (4′,5,7-trihydroxyflavanone)
8.77e4
273
HO
OH
O
OH
O
MW: 272.25 amu
m/z

(5) 3,5,7-Trihydroxy-2-phenylchroman-4-one

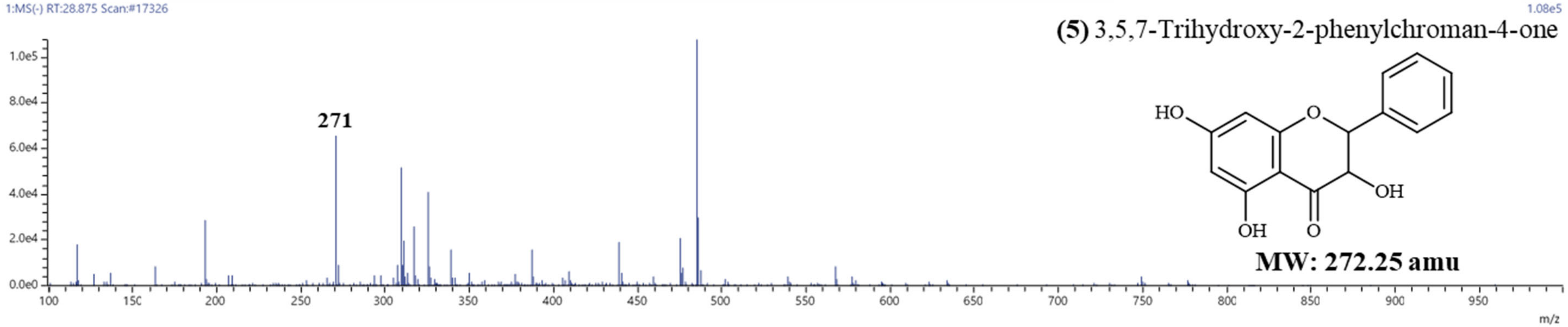

(6) Quersetin (3,5,7,3′,4′-pentahydroxyflavone)

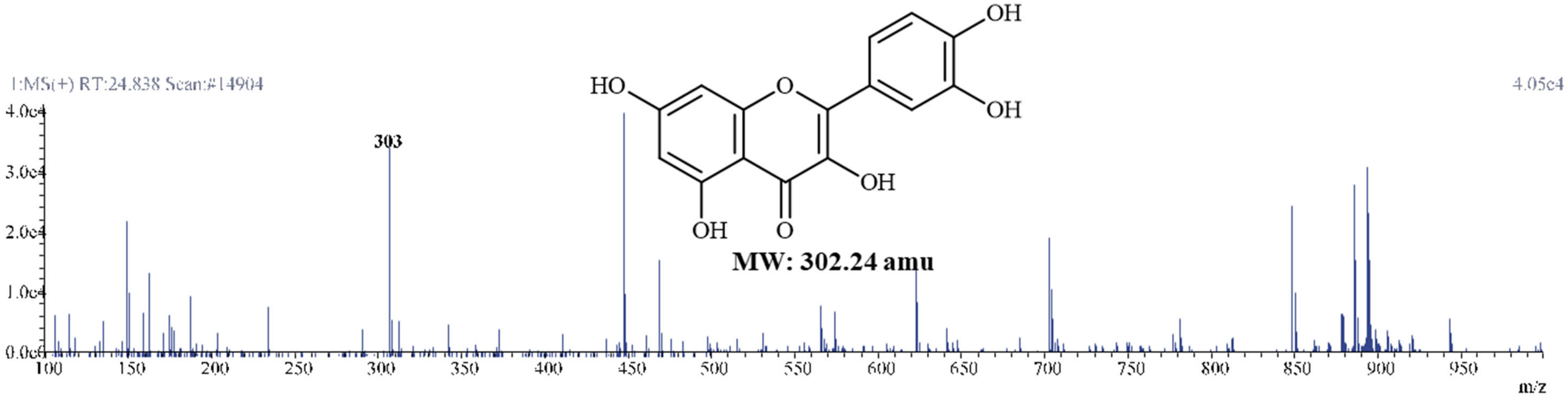

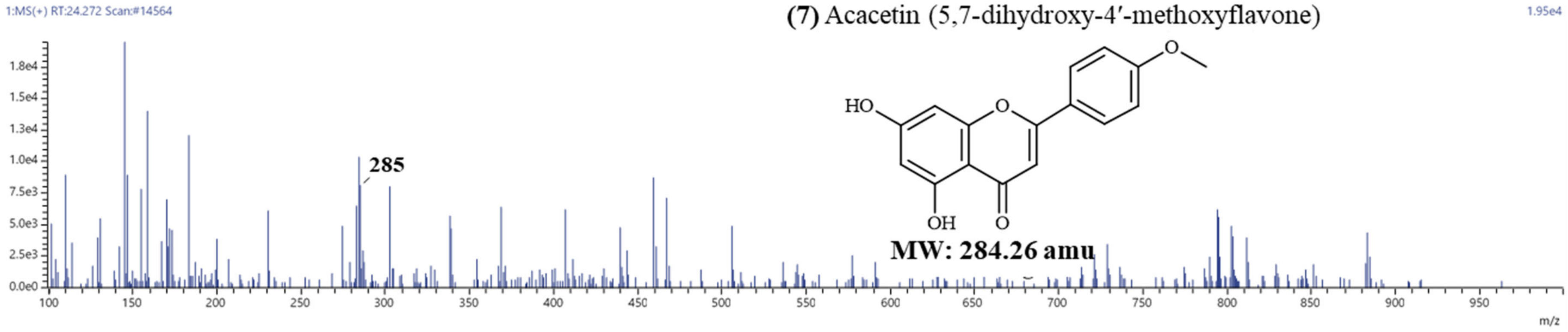
1:MS(+) RT:24.272 Scan:#14564
(7) Acacetin (5,7-dihydroxy-4′-methoxyflavone)
1.95e4
285
HO
O
O
OH
O
MW: 284.26 amu
m/z

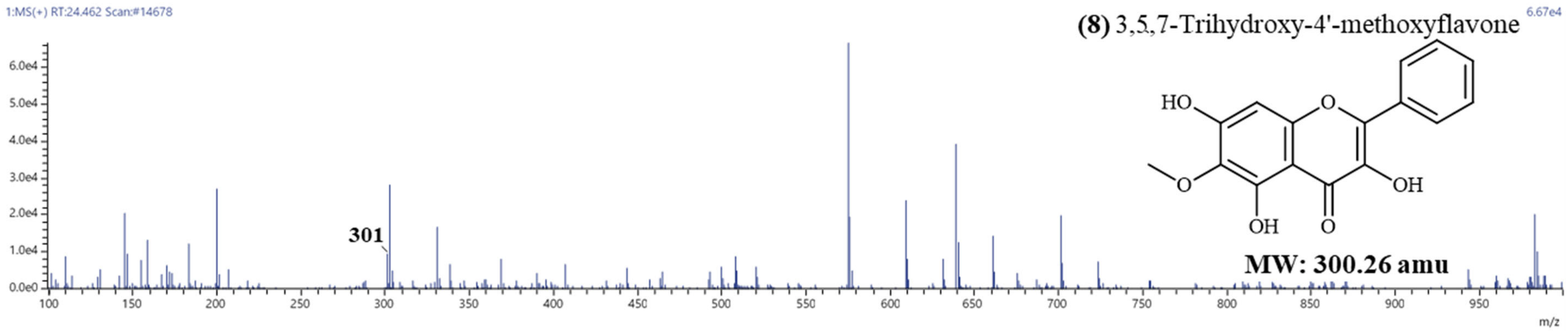
1:MS(+) RT:24.462 Scan:#14678
(8) 3,5,7-Trihydroxy-4'-methoxyflavone
6.67e4
301
HO
O
O
OH
OH
O
MW: 300.26 amu
m/z

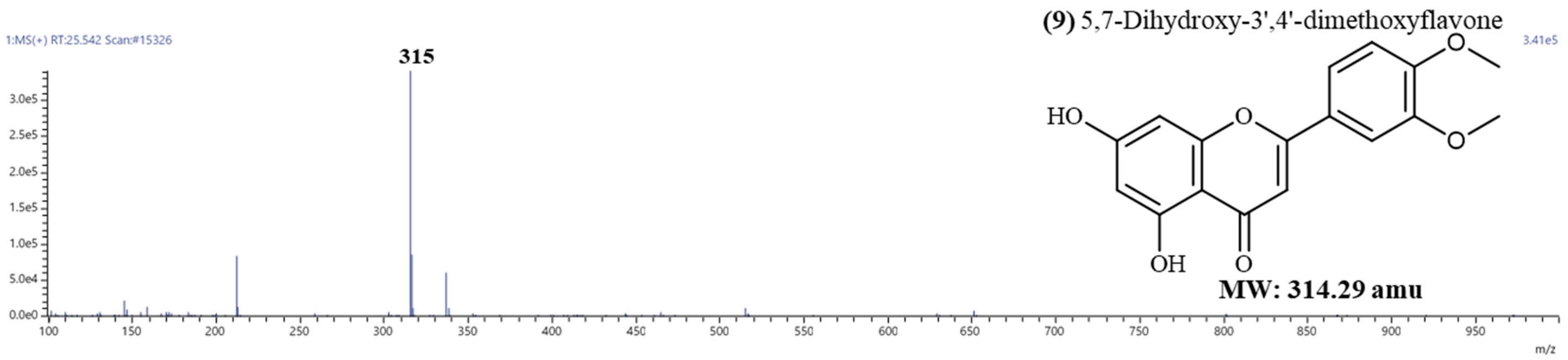
(9) 5,7-Dihydroxy-3',4'-dimethoxyflavone
1:MS(+) RT:25.542 Scan:#15326
3.41e5
315
MW: 314.29 amu
m/z

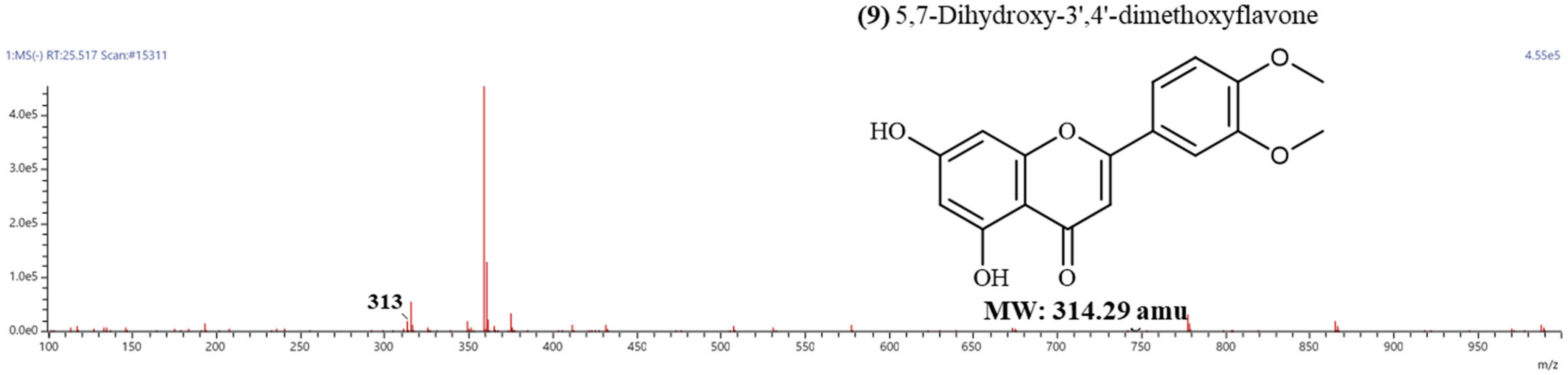
(9) 5,7-Dihydroxy-3',4'-dimethoxyflavone
1:MS(-) RT:25.517 Scan:#15311
4.55e5
313
MW: 314.29 amu
m/z

(10) 5-Hydroxy-6,7,4'-trimethoxyflavanone

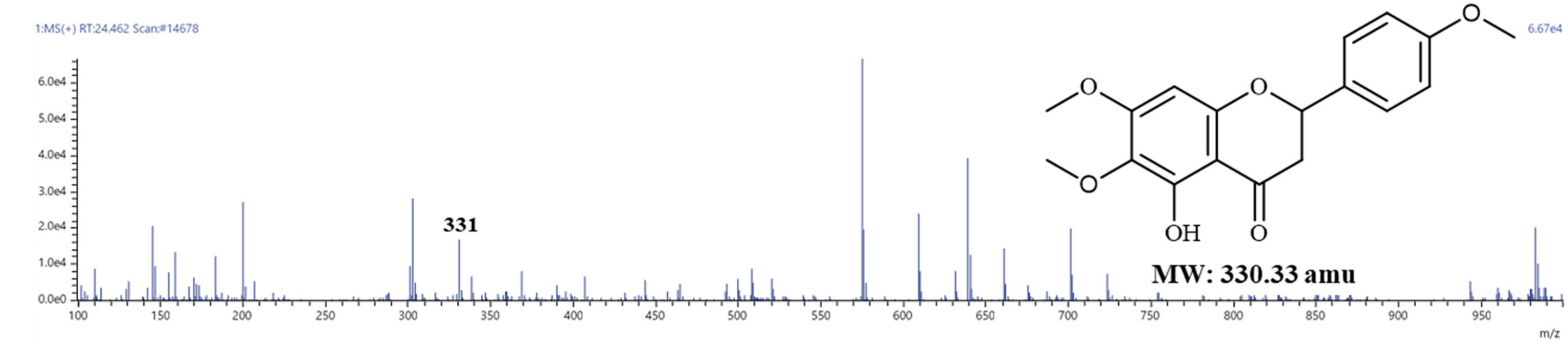

(11) Ombuin (3,3',5-trihydroxy-7,4'-dimethoxyflavone)

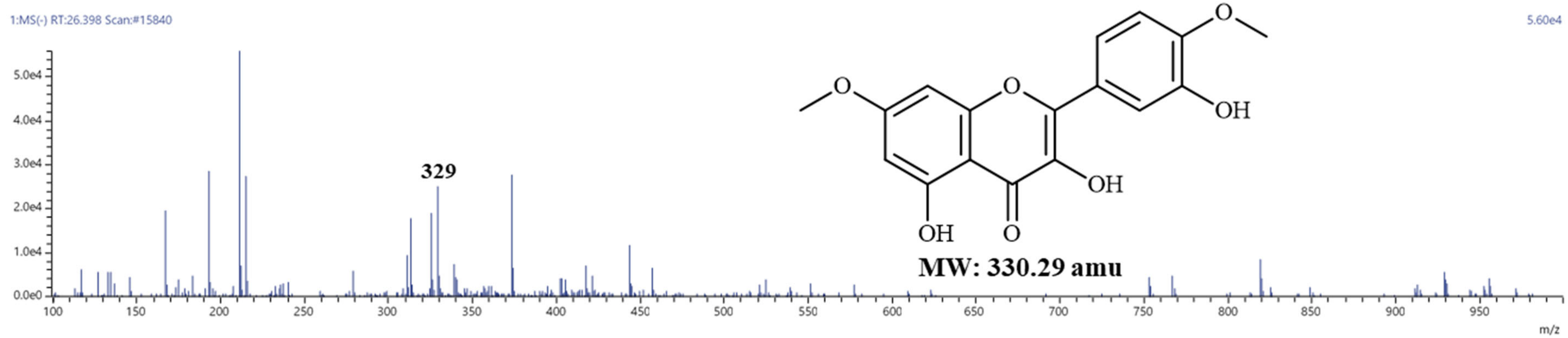

(12) 6-Methoxyluteolin

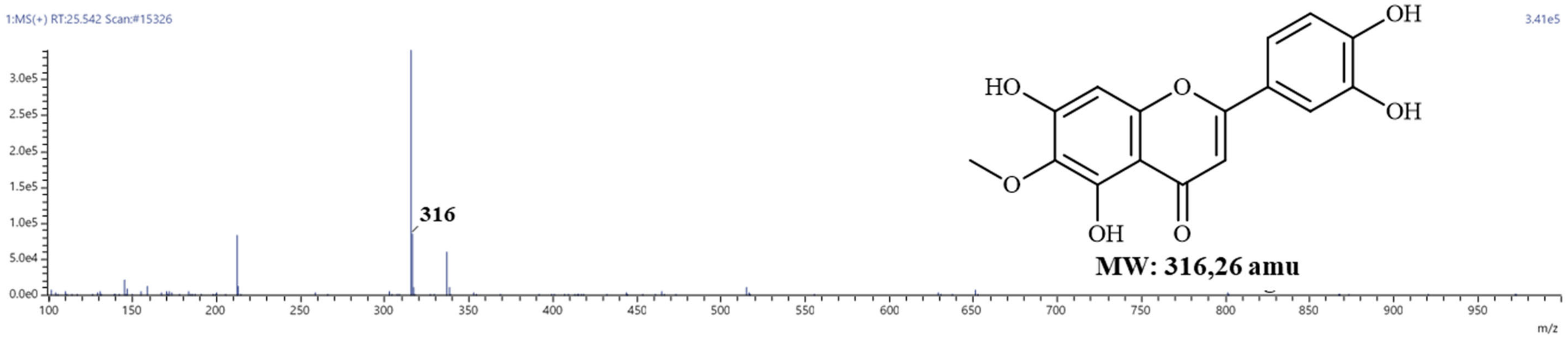

(13) Subscandenin (5,7-dihydroxy-8,4'-dimethoxyflavanone)

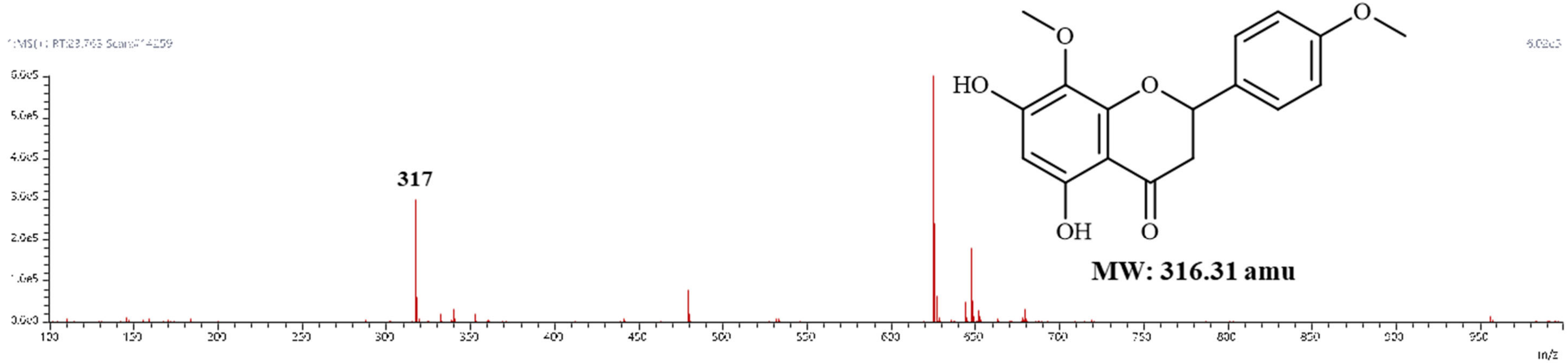

(14) Persikogenin (5,3'-dihydroxy-7,4'-dimethoxyflavanone)

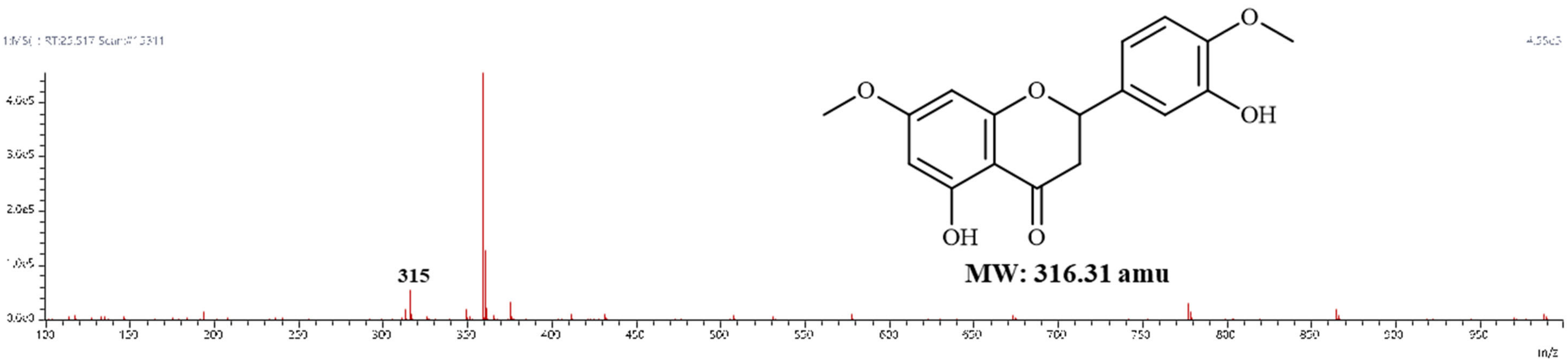

(15) Isoquercitrin (quercetin-3-O-glucopyranoside)

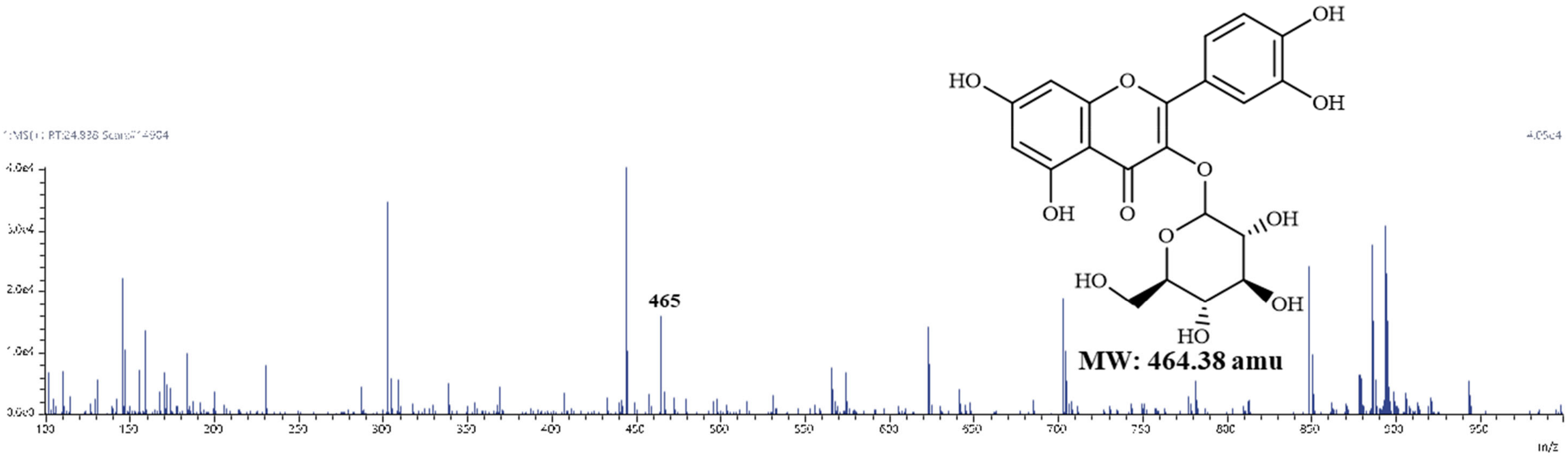

(16) Odoratin (2-hydroxy-4',5',6,4-tetramethoxy chalcone)

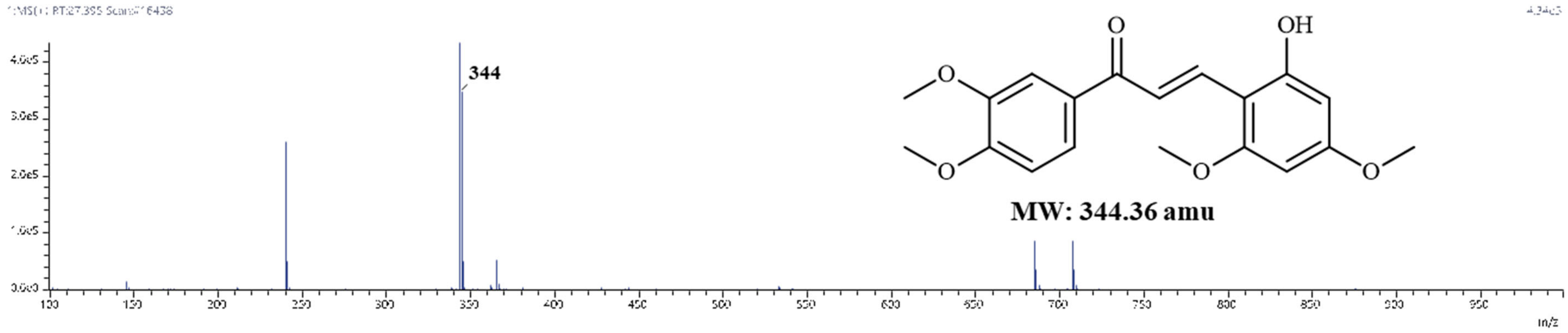

(16) Odoratin (2-hydroxy-4',5',6,4-tetramethoxy chalcone)

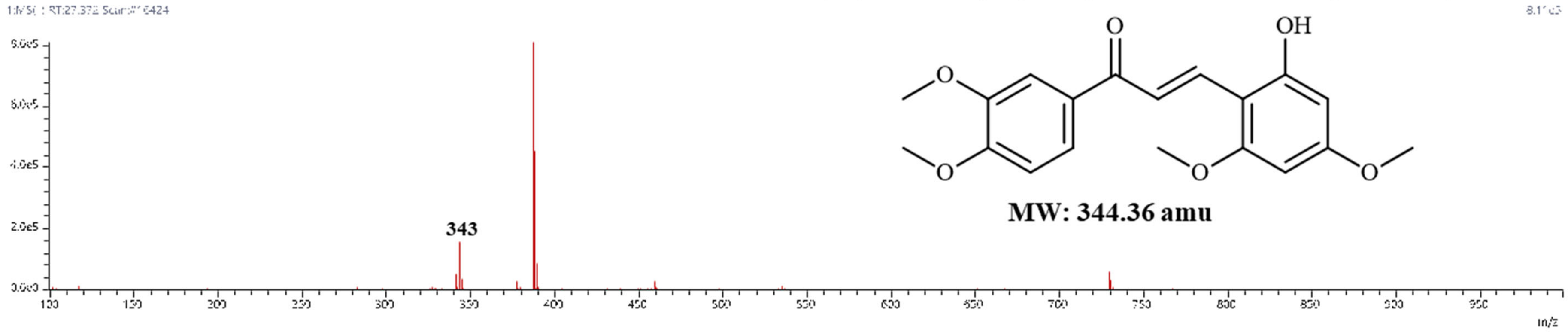

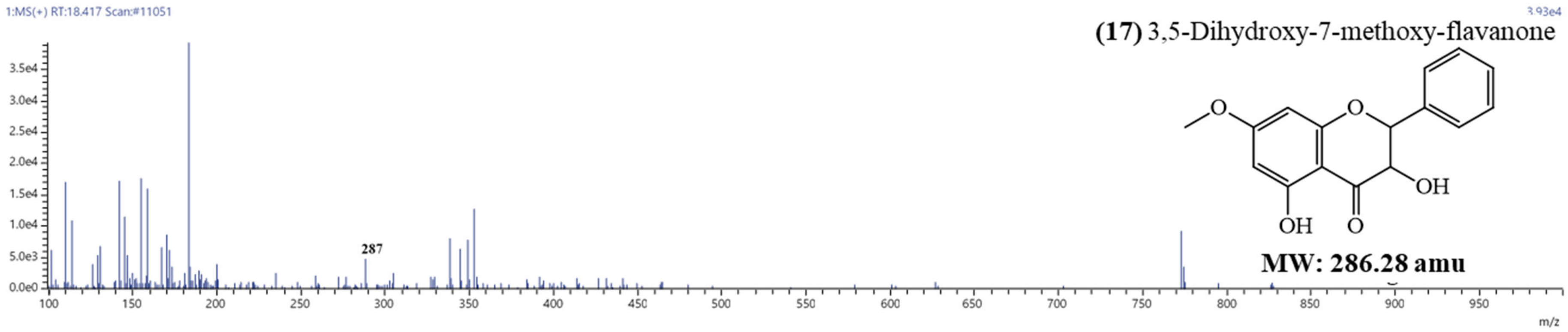
1:MS(+) RT:18.417 Scan:#11051
3.93e4
(17) 3,5-Dihydroxy-7-methoxy-flavanone
OH
OH
O
O
MW: 286.28 amu
287
m/z

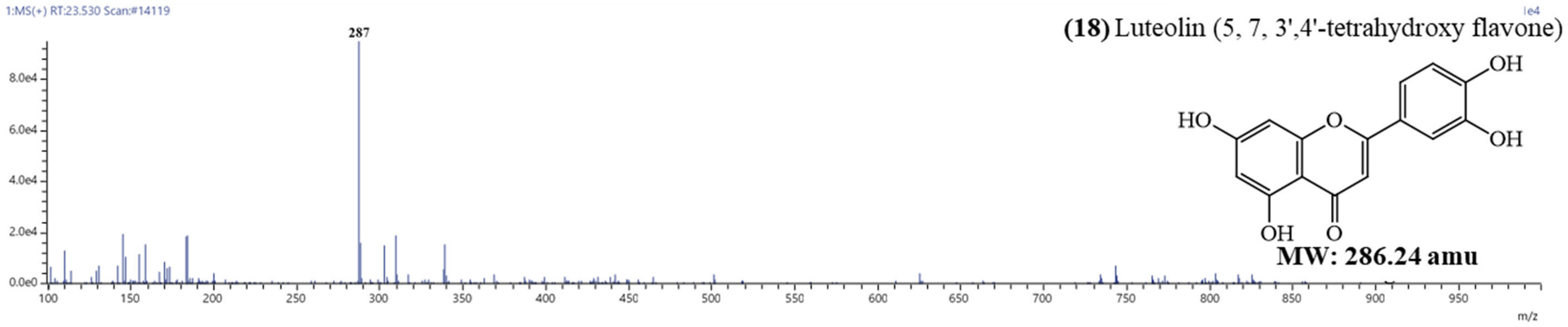
1:MS(+) RT:23.530 Scan:#14119
1e4
(18) Luteolin (5, 7, 3',4'-tetrahydroxy flavone)
HO
OH
OH
OH
O
O
MW: 286.24 amu
287
m/z

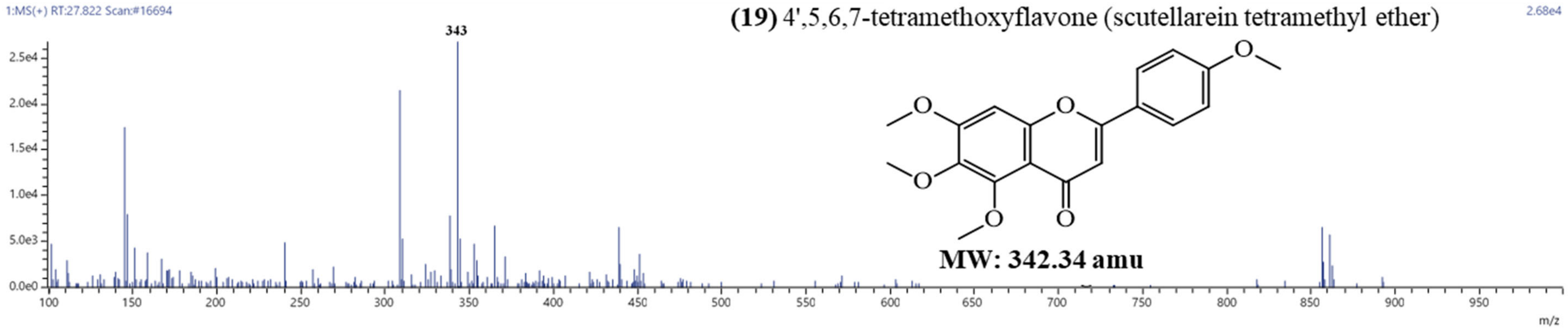
1:MS(+) RT:27.822 Scan:#16694
(19) 4',5,6,7-tetramethoxyflavone (scutellarein tetramethyl ether)
2.68e4
343
MW: 342.34 amu
m/z

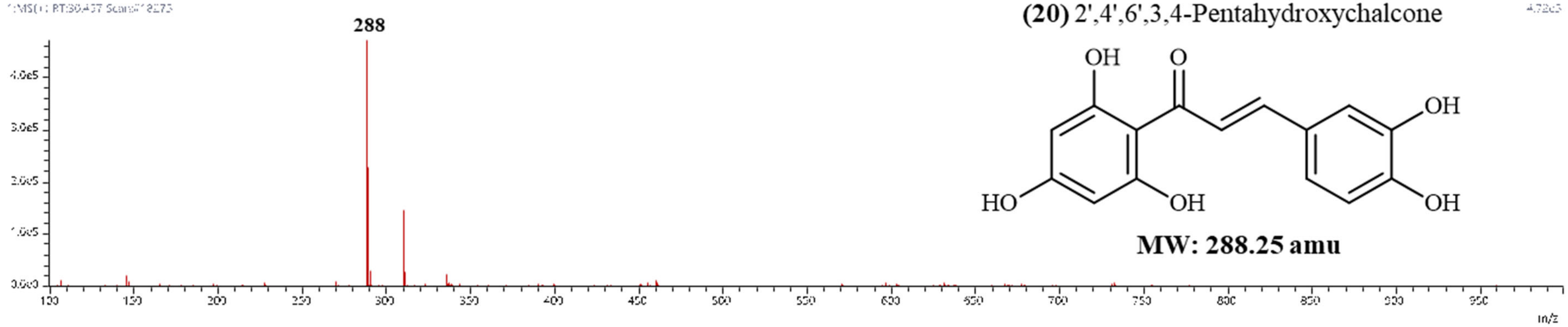
(20) 2',4',6',3,4-Pentahydroxychalcone
288
OH
O
OH
HO
OH
OH
MW: 288.25 amu

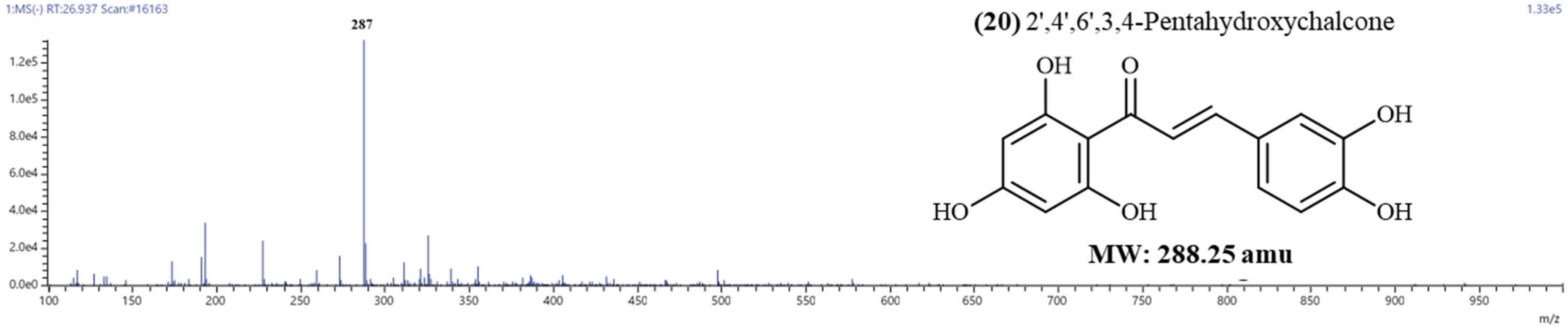
1:MS(-) RT:26.937 Scan:#16163
1.33e5
287
(20) 2',4',6',3,4-Pentahydroxychalcone
OH
O
OH
HO
OH
OH
MW: 288.25 amu
m/z

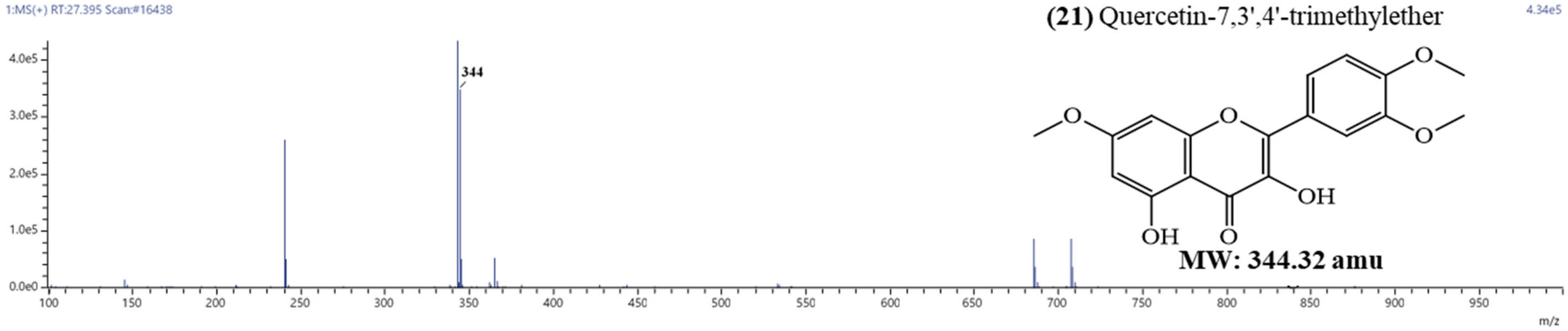
1:MS(+) RT:27.395 Scan:#16438
4.34e5
344
(21) Quercetin-7,3',4'-trimethylether
O
O
O
O
OH
OH
O
MW: 344.32 amu
m/z

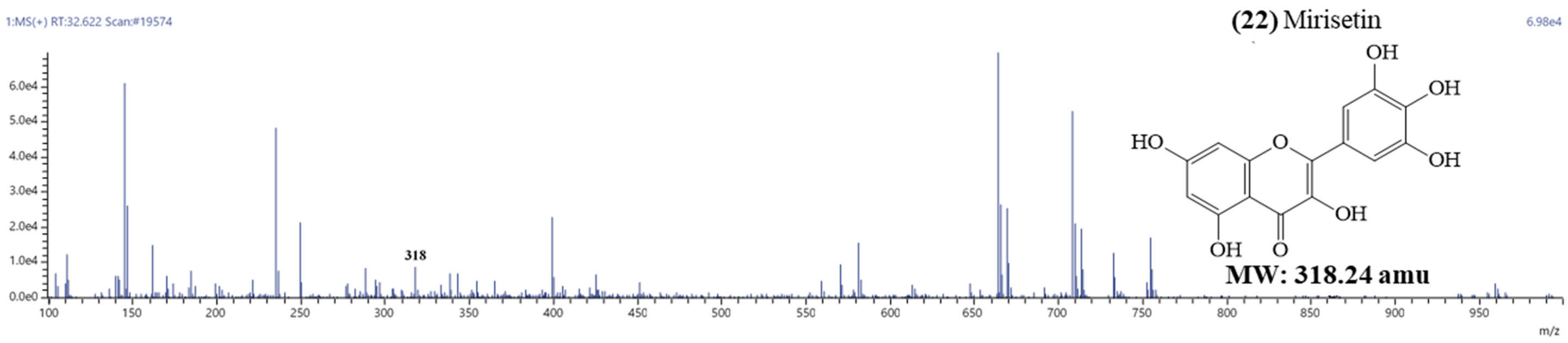
1:MS(+) RT:32.622 Scan:#19574
6.98e4
(22) Mirisetin
318
MW: 318.24 amu
m/z

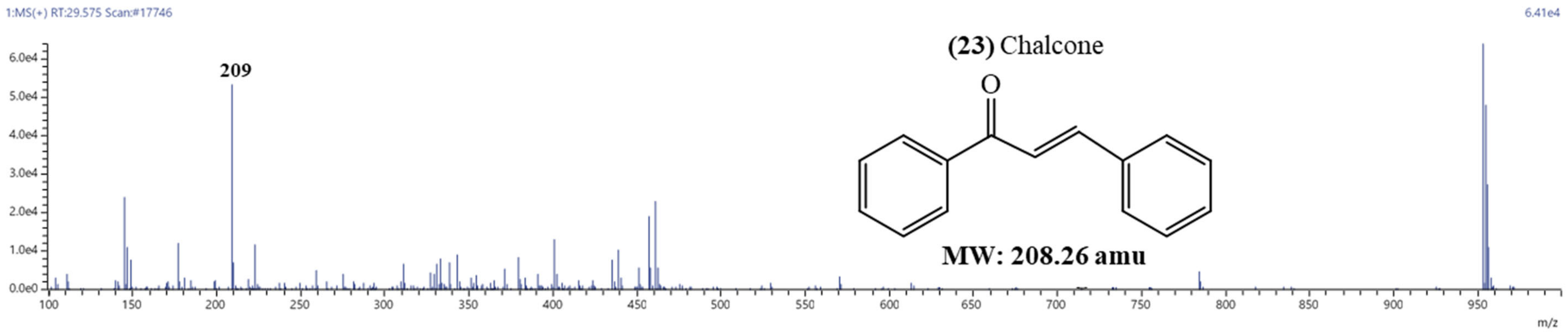
1:MS(+) RT:29.575 Scan:#17746
6.41e4
(23) Chalcone
209
MW: 208.26 amu
m/z

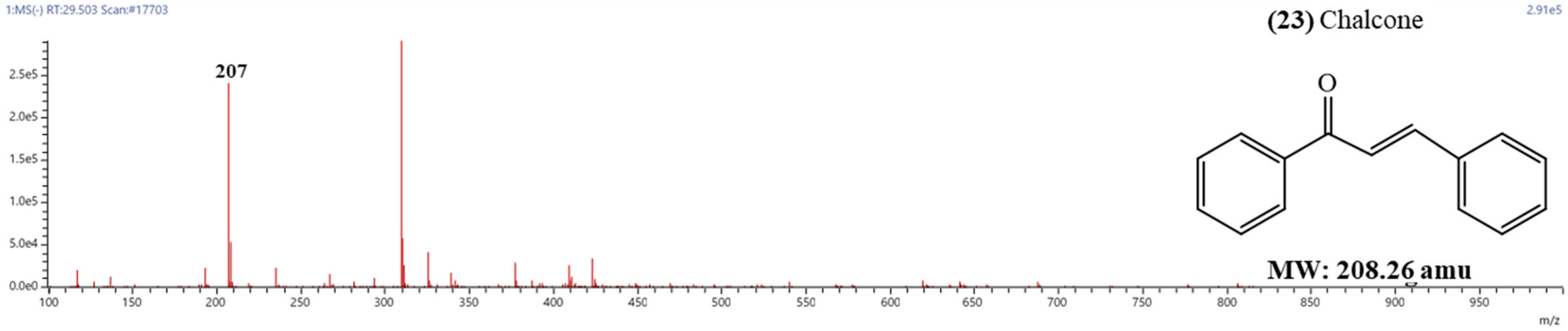

1:MS(-) RT:29.503 Scan:#17703
(23) Chalcone
2.91e5
207
O
MW: 208.26 amu
m/z

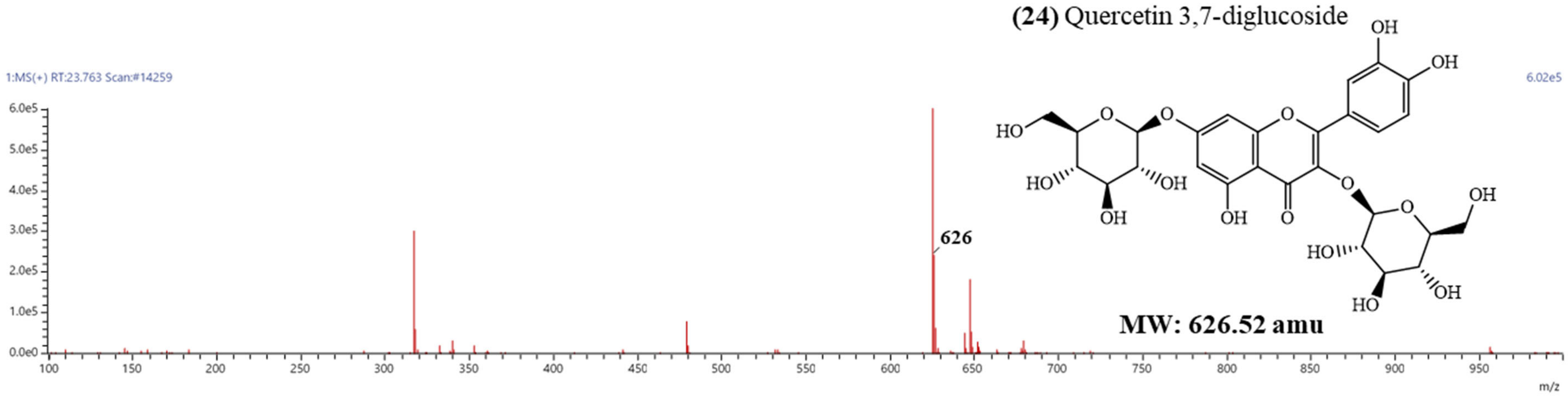

(24) Quercetin 3,7-diglucoside
1:MS(+) RT:23.763 Scan:#14259
6.02e5
626
MW: 626.52 amu
m/z

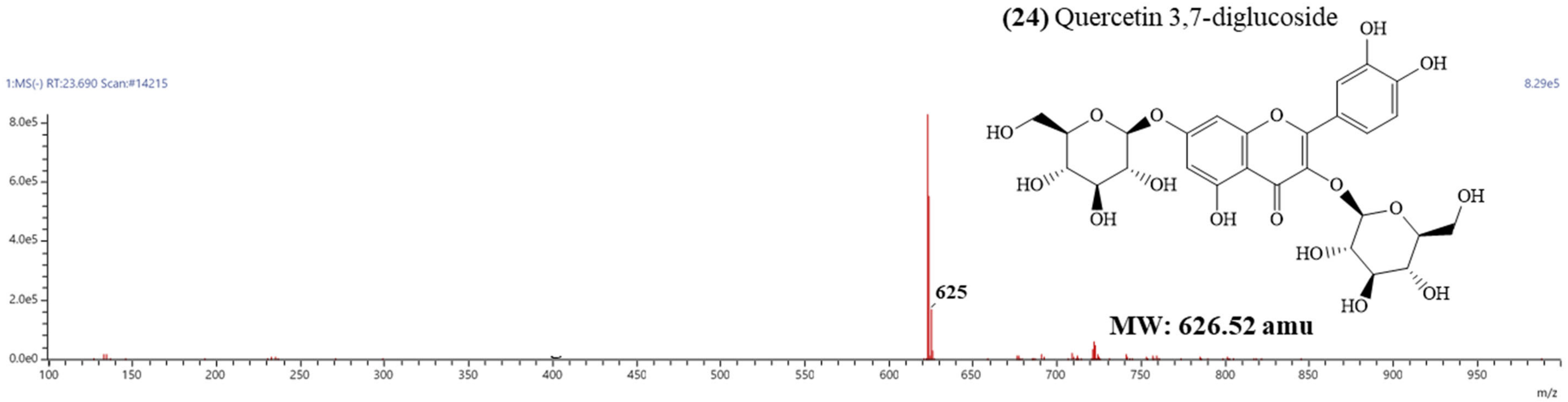
(24) Quercetin 3,7-diglucoside
1:MS(-) RT:23.690 Scan:#14215
8.29e5
625
MW: 626.52 amu
m/z

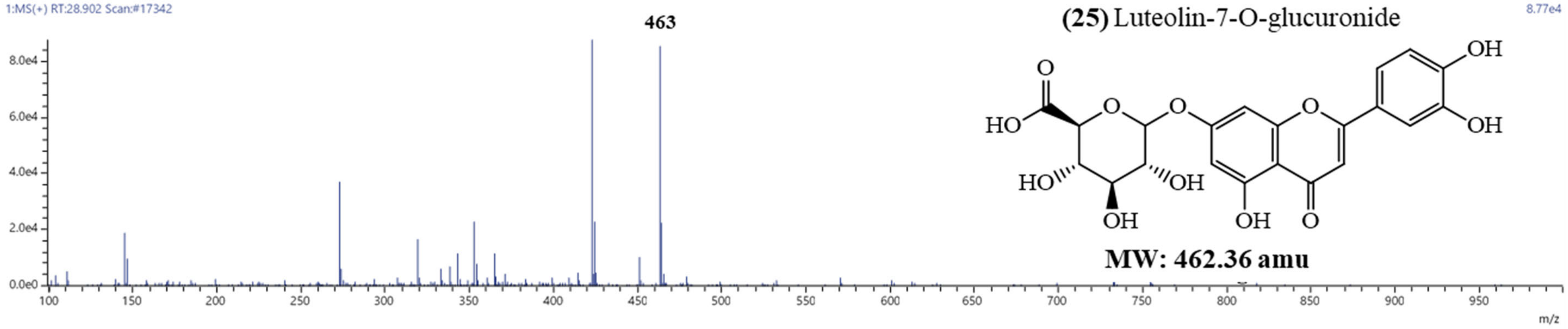
(25) Luteolin-7-O-glucuronide
1:MS(+) RT:28.902 Scan:#17342
8.77e4
463
MW: 462.36 amu
m/z

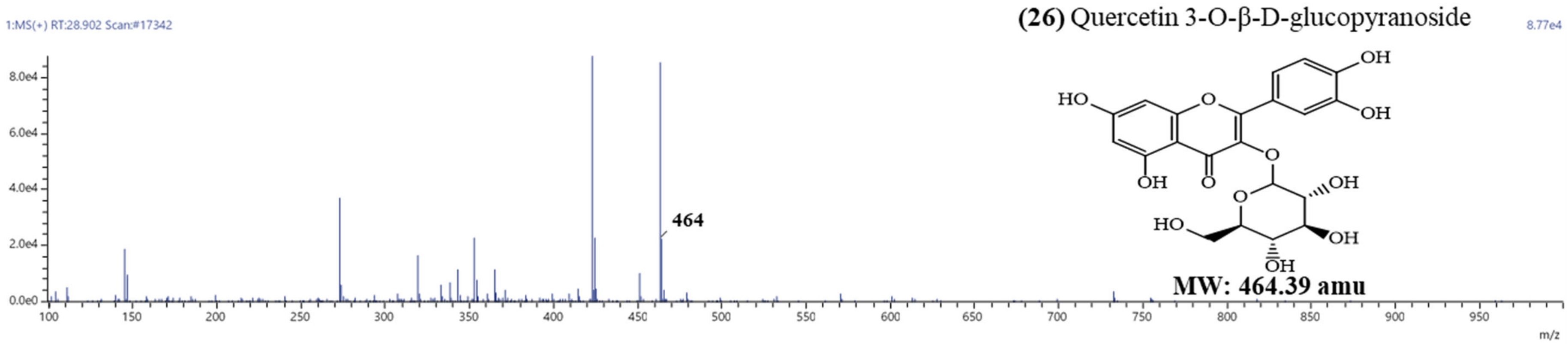
1:MS(+) RT:28.902 Scan:#17342
(26) Quercetin 3-O-β-D-glucopyranoside
8.77e4
464
MW: 464.39 amu
m/z

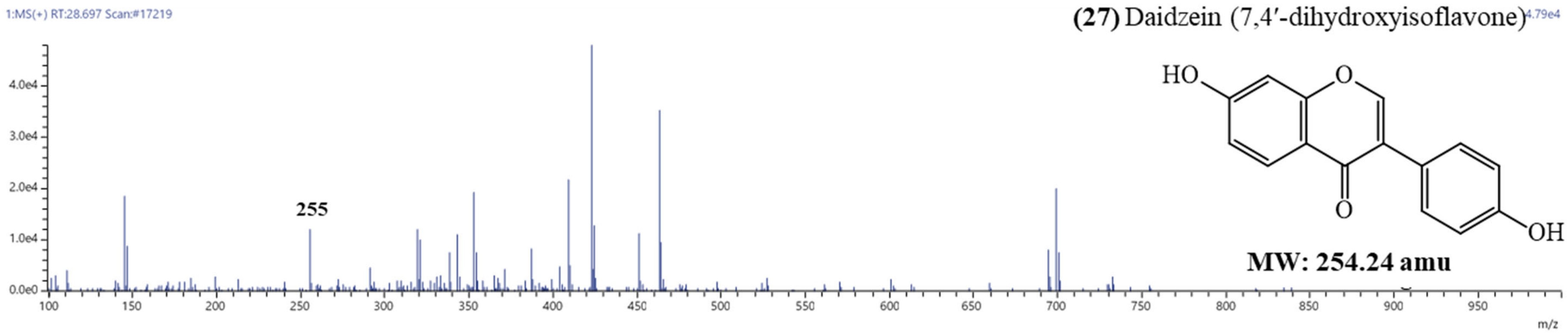
1:MS(+) RT:28.697 Scan:#17219
(27) Daidzein (7,4′-dihydroxyisoflavone)
4.79e4
255
MW: 254.24 amu
m/z

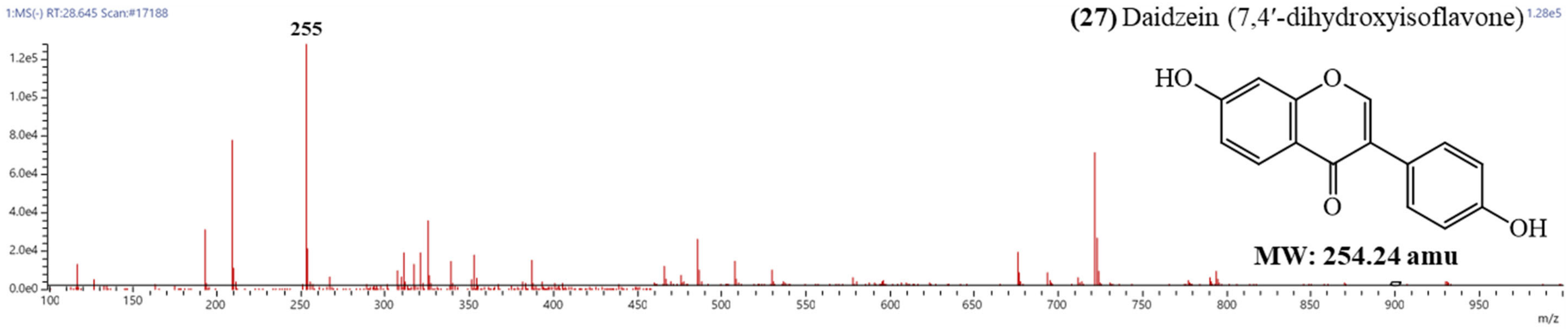
1:MS(-) RT:28.645 Scan:#17188
255
(27) Daidzein (7,4′-dihydroxyisoflavone)
1.28e5
HO
O
O
OH
MW: 254.24 amu
m/z

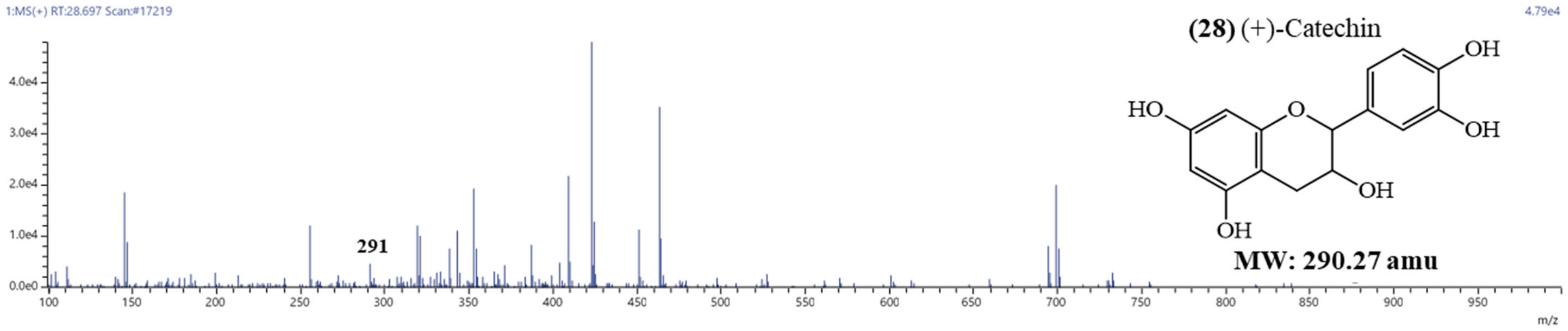
1:MS(+) RT:28.697 Scan:#17219
4.79e4
(28) (+)-Catechin
291
HO
O
OH
OH
OH
OH
MW: 290.27 amu
m/z

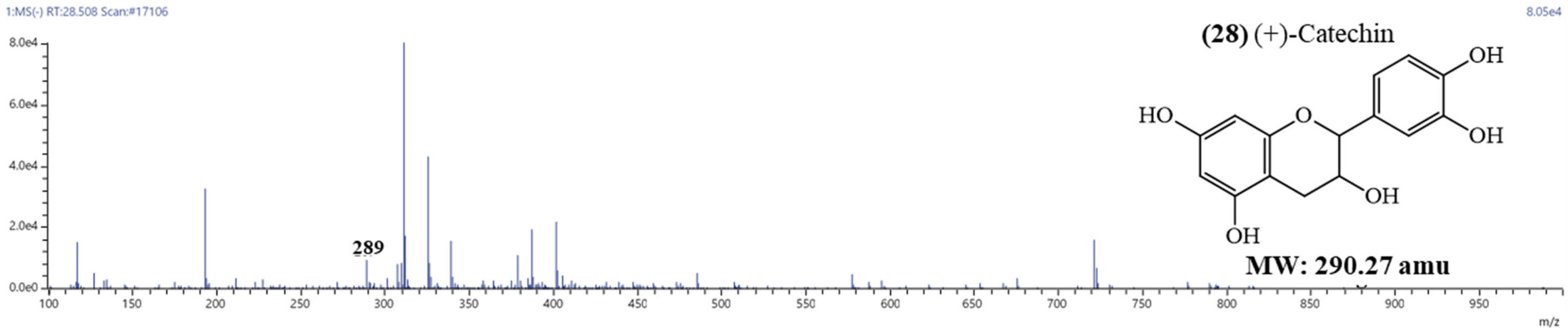
1:MS(-) RT:28.508 Scan:#17106
8.05e4
(28) (+)-Catechin
HO
O
OH
OH
OH
OH
289
MW: 290.27 amu
m/z

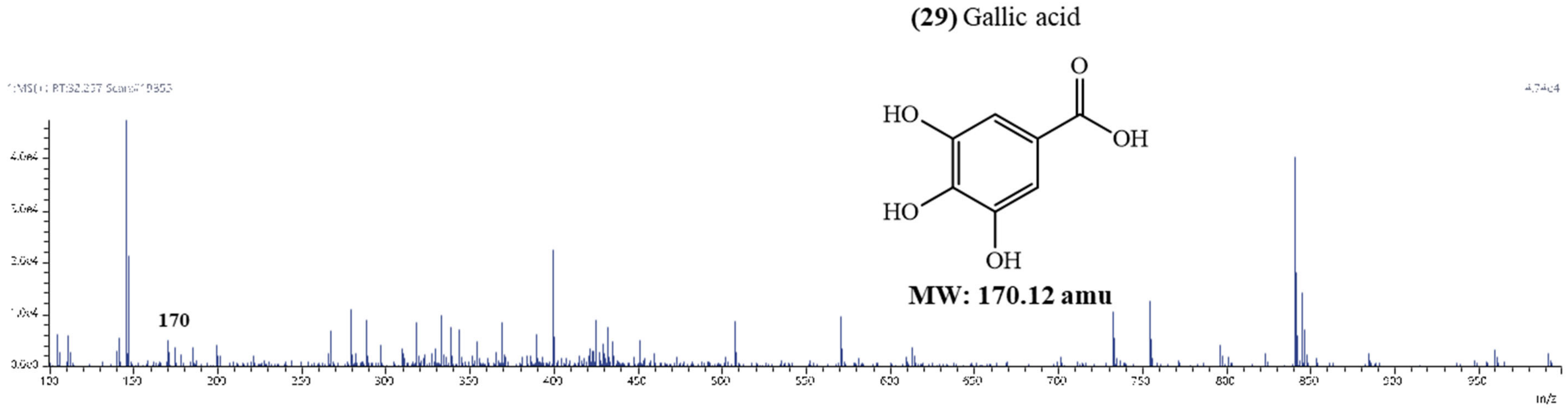
(29) Gallic acid
O
HO
OH
HO
OH
170
MW: 170.12 amu
m/z

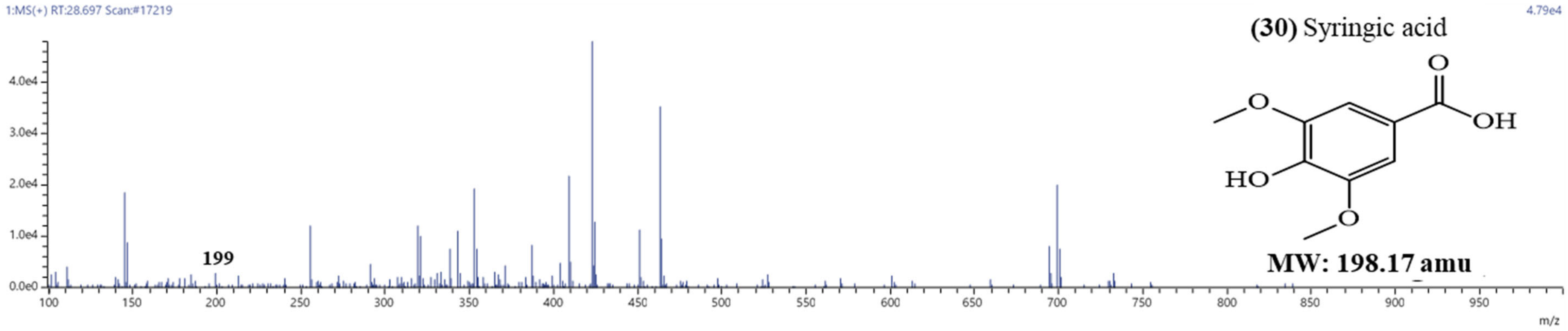
1:MS(+) RT:28.697 Scan:#17219
4.79e4
(30) Syringic acid
O
O
OH
HO
O
MW: 198.17 amu
199
4.0e4
3.0e4
2.0e4
1.0e4
0.0e0
100 150 200 250 300 350 400 450 500 550 600 650 700 750 800 850 900 950
m/z

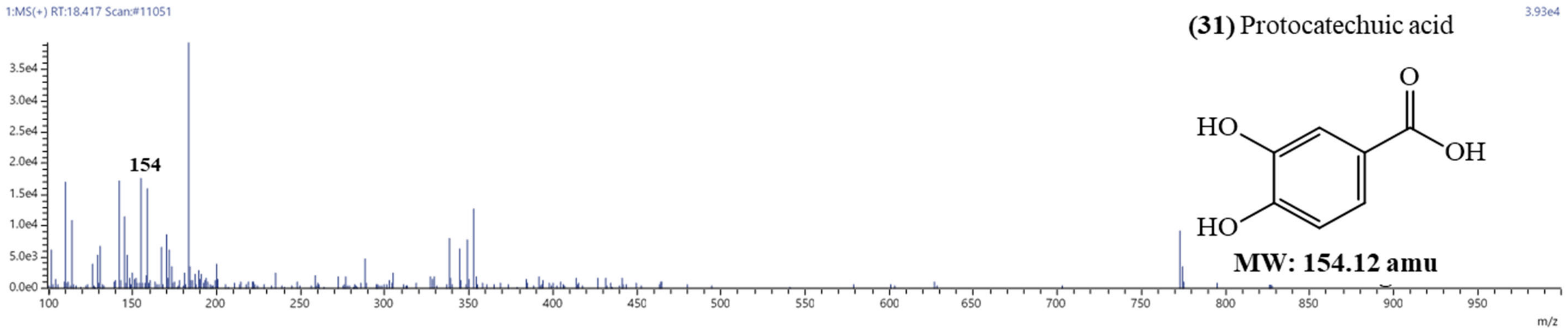
1:MS(+) RT:18.417 Scan:#11051
3.93e4
(31) Protocatechuic acid
O
HO
OH
HO
MW: 154.12 amu
154
3.5e4
3.0e4
2.5e4
2.0e4
1.5e4
1.0e4
5.0e3
0.0e0
100 150 200 250 300 350 400 450 500 550 600 650 700 750 800 850 900 950
m/z

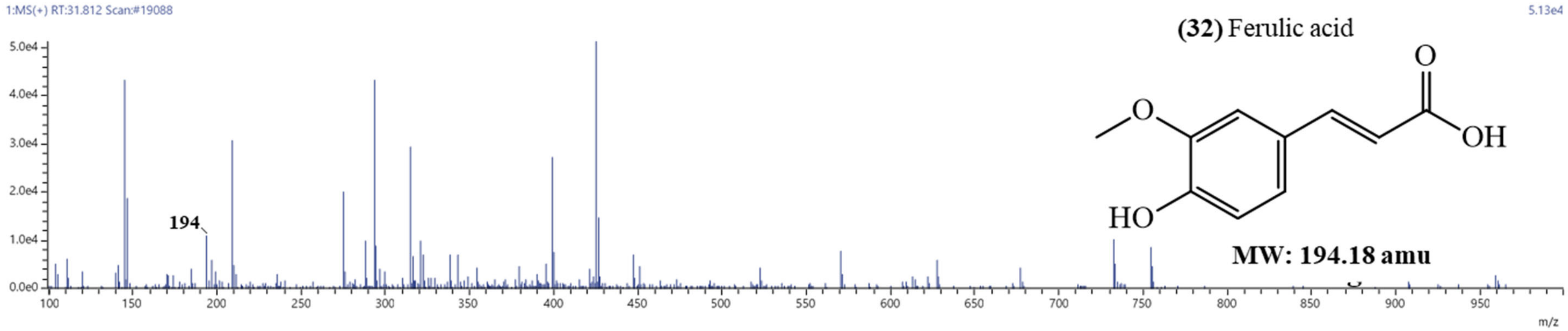
1:MS(+) RT:31.812 Scan:#19088
5.13e4
(32) Ferulic acid
O
OH
HO
MW: 194.18 amu
194
m/z

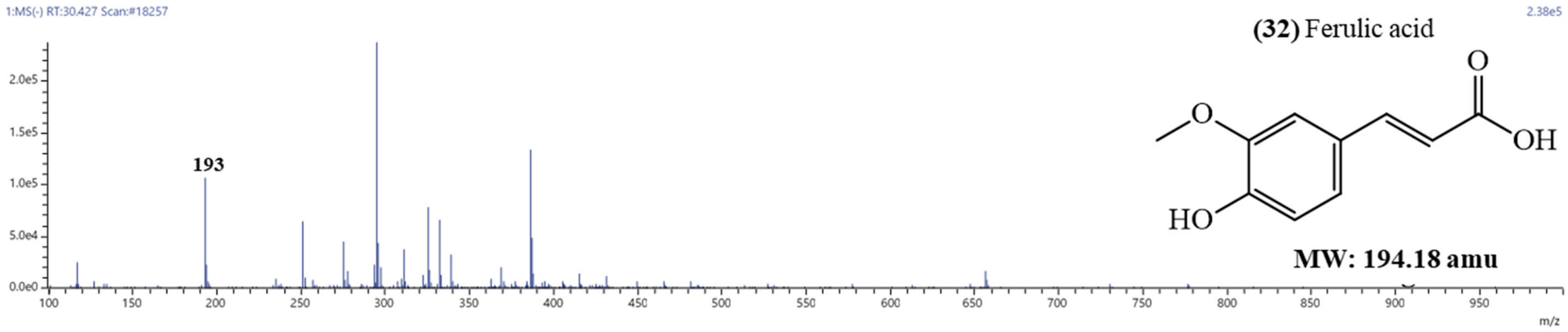
1:MS(-) RT:30.427 Scan:#18257
2.38e5
(32) Ferulic acid
O
OH
HO
MW: 194.18 amu
193
m/z

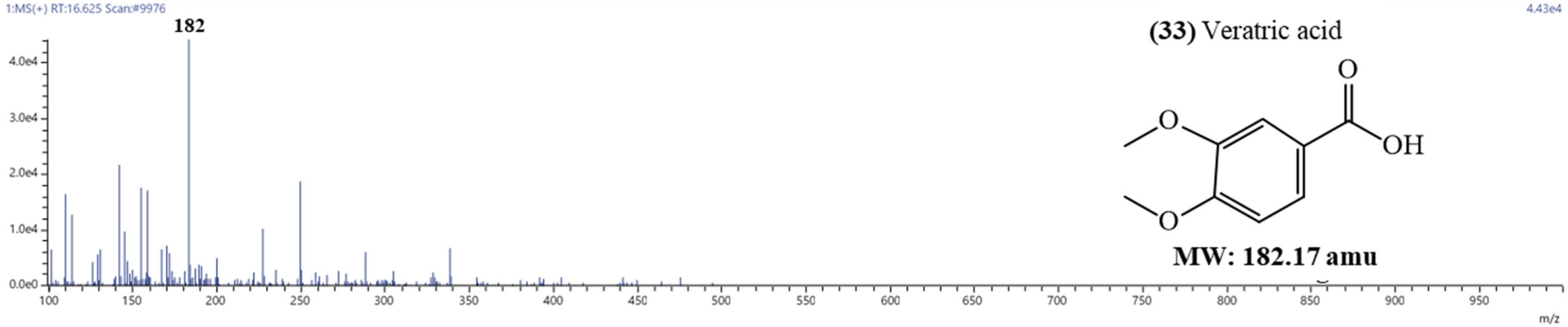
1:MS(+) RT:16.625 Scan:#9976
4.43e4
182
(33) Veratric acid
O
OH
O
O
MW: 182.17 amu
m/z

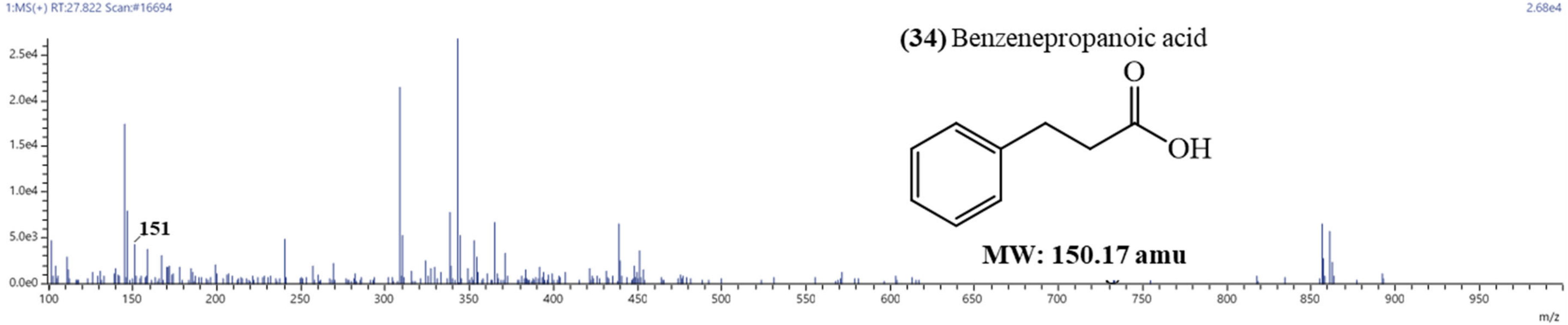
1:MS(+) RT:27.822 Scan:#16694
2.68e4
151
(34) Benzenepropanoic acid
O
OH
MW: 150.17 amu
m/z

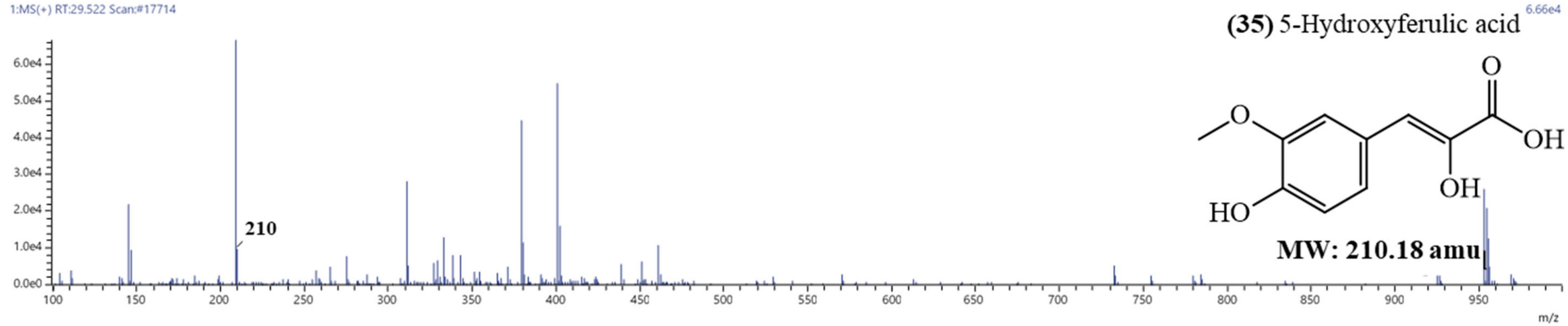

1:MS(+) RT:29.522 Scan:#17714
6.66e4
(35) 5-Hydroxyferulic acid
210
MW: 210.18 amu
m/z

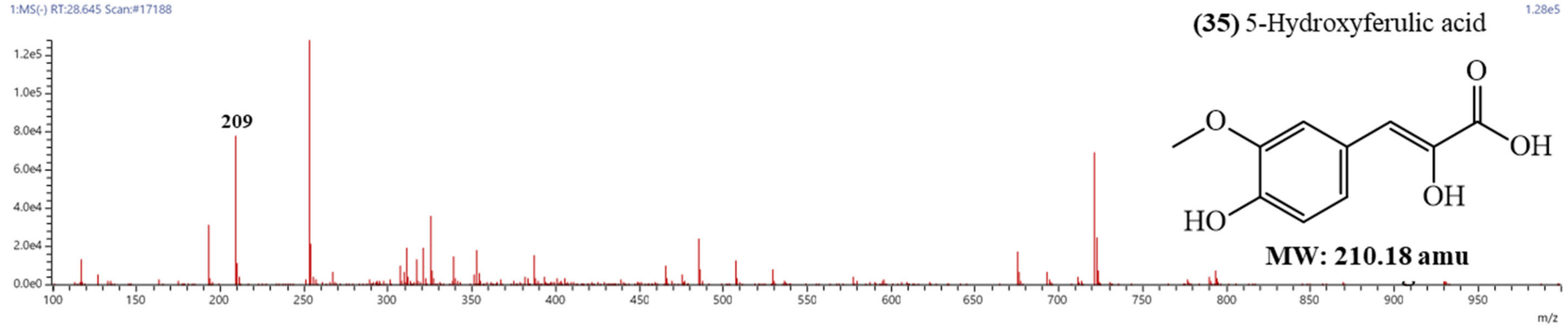

1:MS(-) RT:28.645 Scan:#17188
1.28e5
(35) 5-Hydroxyferulic acid
209
MW: 210.18 amu
m/z

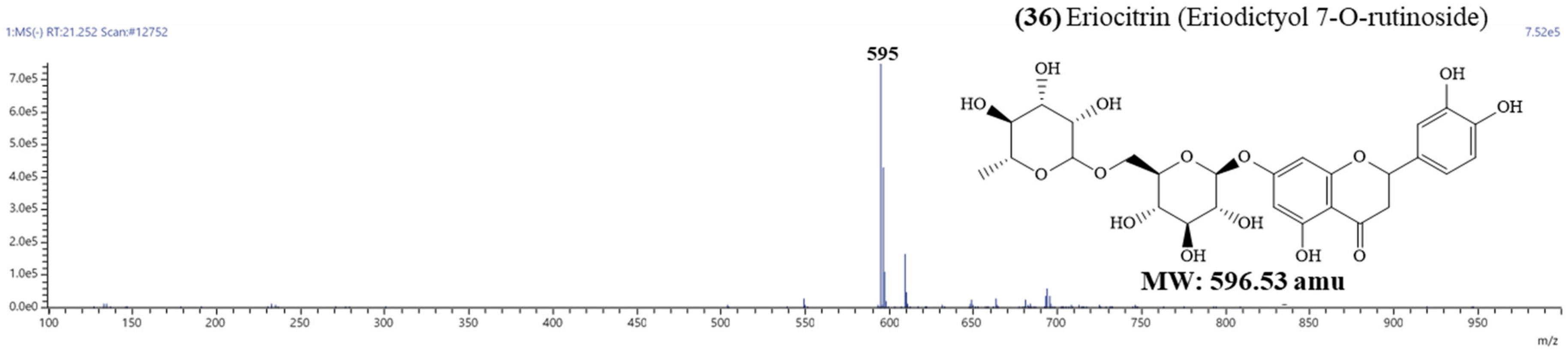

1:MS(-) RT:21.252 Scan:#12752
7.52e5
595
(36) Eriocitrin (Eriodictyol 7-O-rutinoside)
MW: 596.53 amu
m/z

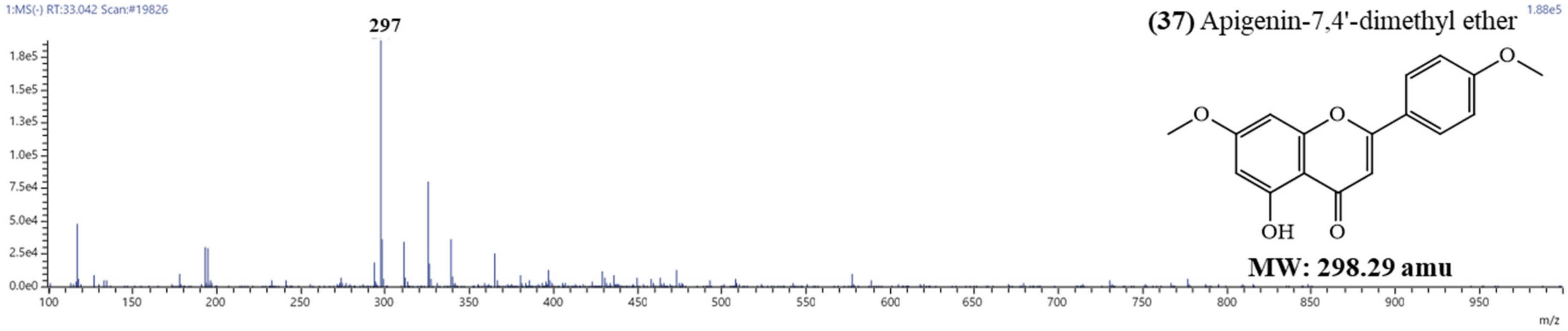

1:MS(-) RT:33.042 Scan:#19826
1.88e5
297
(37) Apigenin-7,4'-dimethyl ether
MW: 298.29 amu
m/z

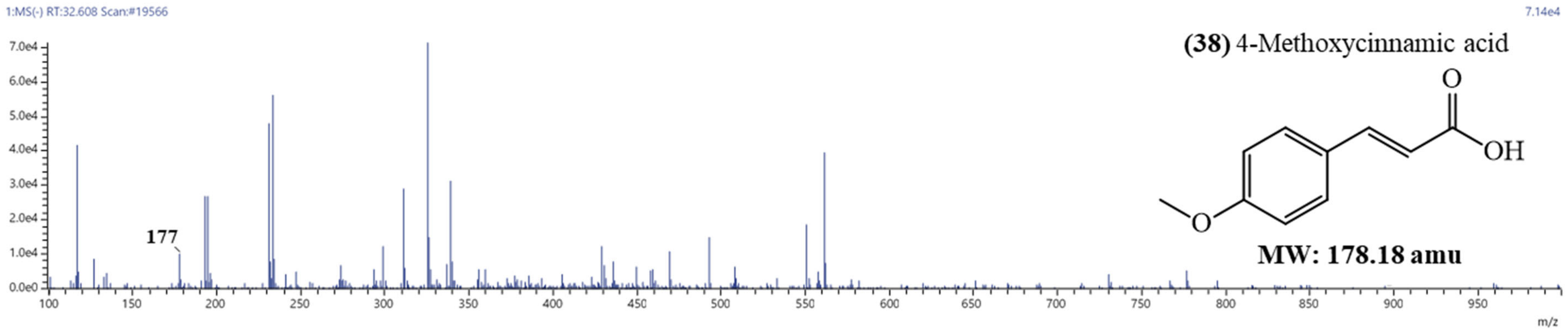
1:MS(-) RT:32.608 Scan:#19566
7.14e4
(38) 4-Methoxycinnamic acid
MW: 178.18 amu
177
m/z

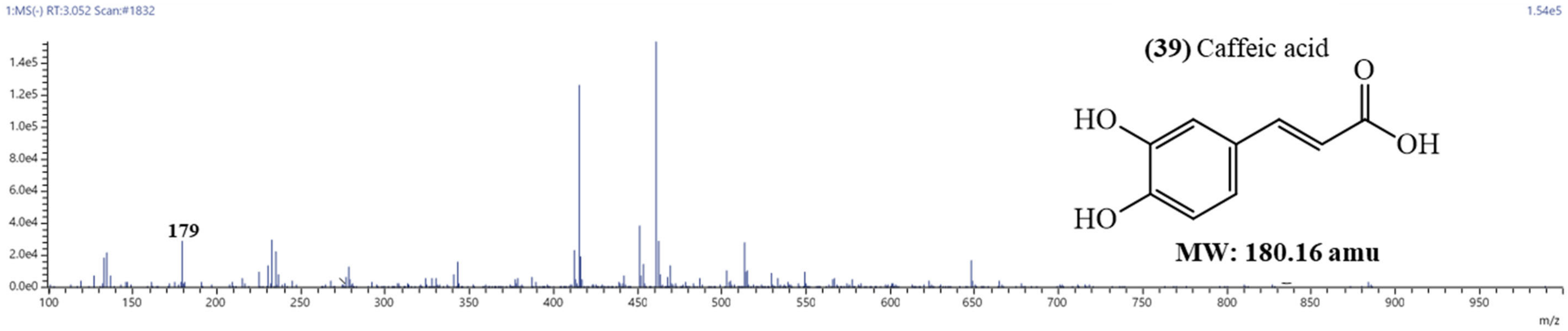
1:MS(-) RT:3.052 Scan:#1832
1.54e5
(39) Caffeic acid
MW: 180.16 amu
179
m/z

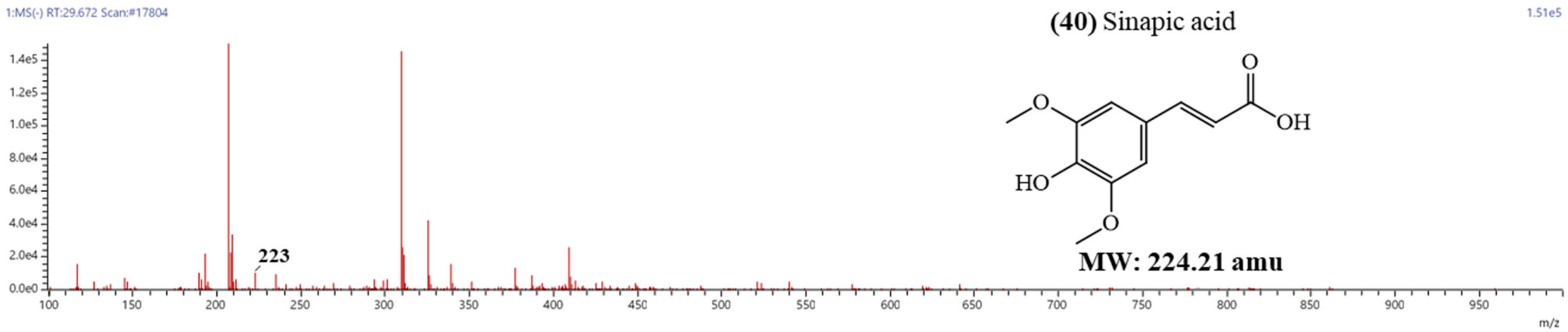
1:MS(-) RT:29.672 Scan:#17804
1.51e5
(40) Sinapic acid
223
MW: 224.21 amu
m/z

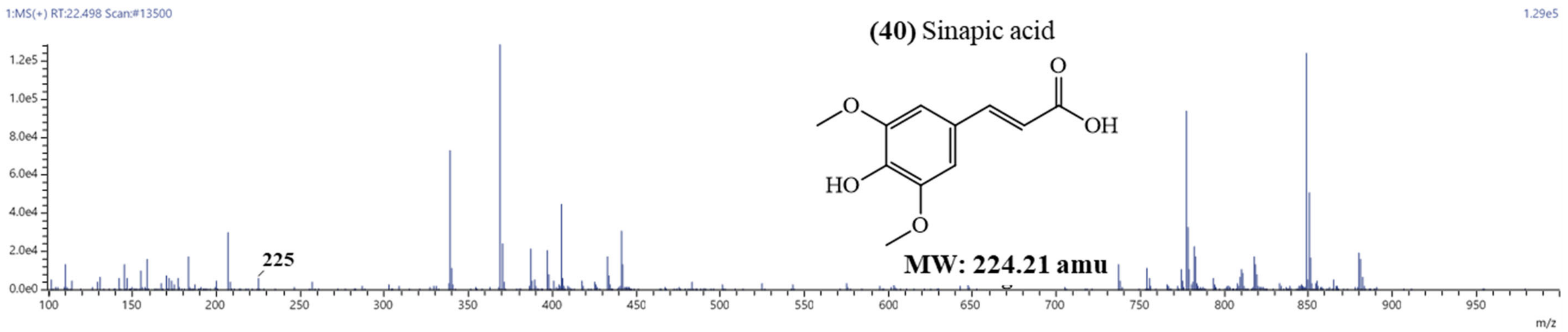
1:MS(+) RT:22.498 Scan:#13500
1.29e5
(40) Sinapic acid
225
MW: 224.21 amu
m/z

(41) Myricetin 3-β-D-glucopyranoside

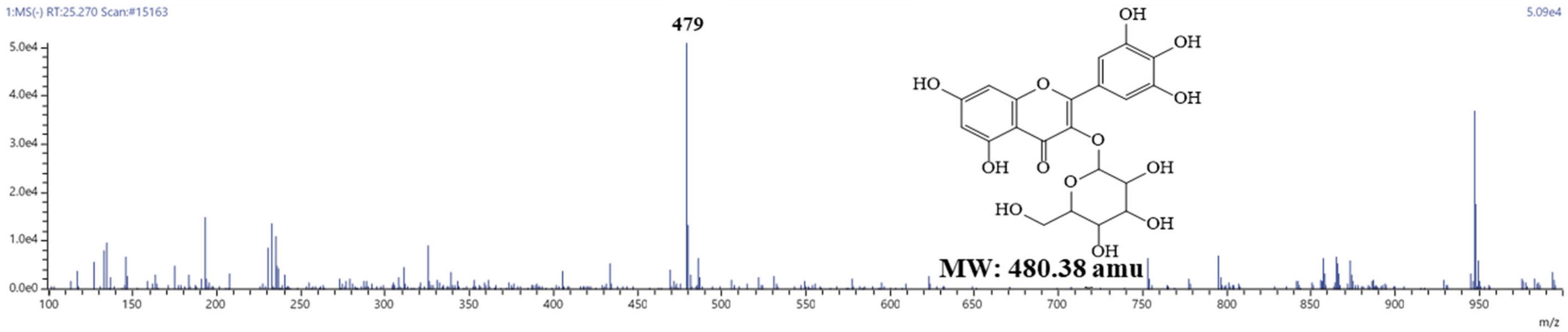

(42) Kaempferol-4′-glucoside

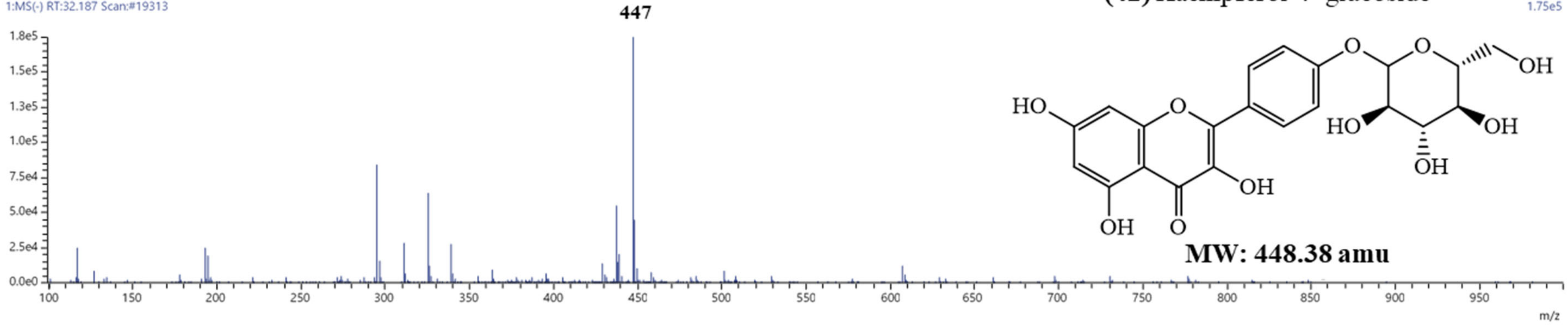

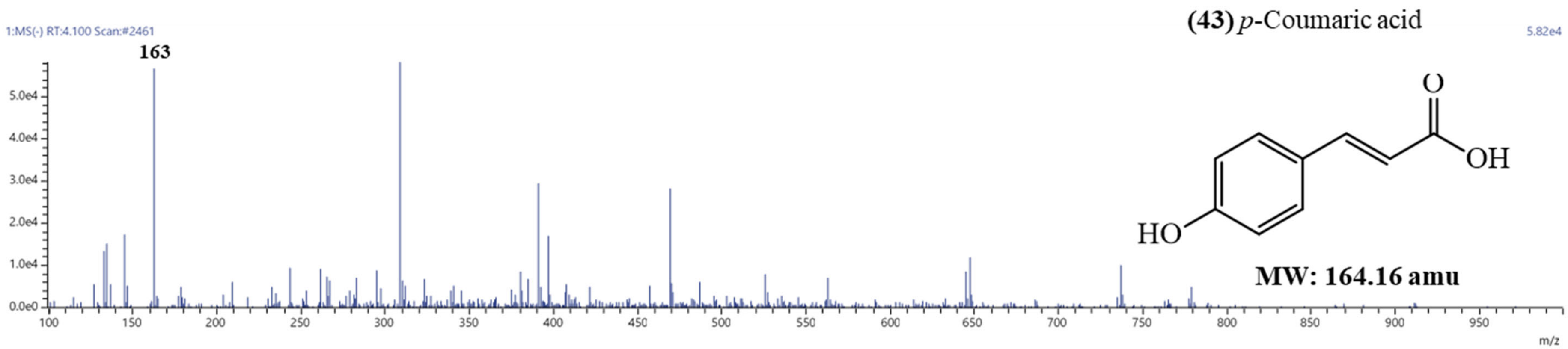
1:MS(-) RT:4.100 Scan:#2461
5.82e4
(43) p-Coumaric acid
163
MW: 164.16 amu
HO
OH
O
m/z

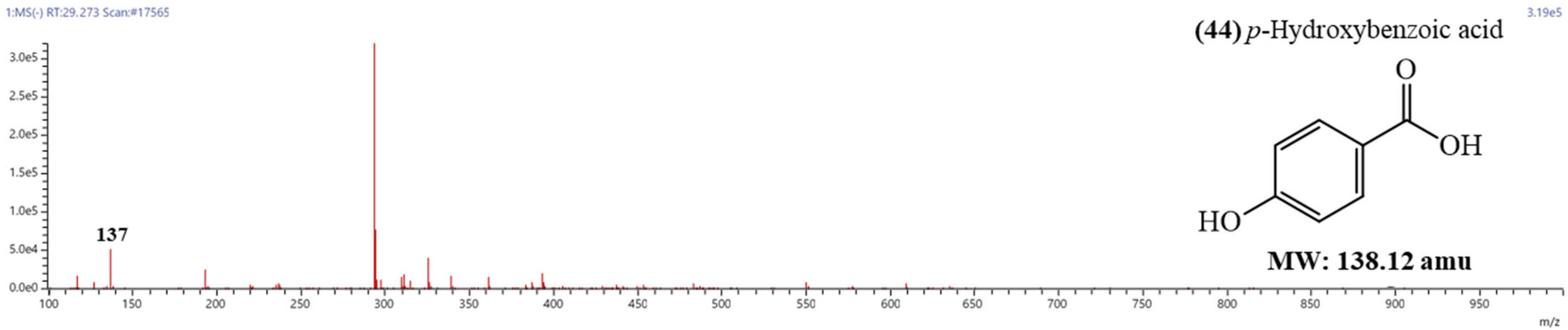
1:MS(-) RT:29.273 Scan:#17565
3.19e5
(44) p-Hydroxybenzoic acid
137
MW: 138.12 amu
HO
OH
O
m/z

(45) 3-phenyl-1-(2,4,6-trihydroxyphenyl)prop-2-en-1-one

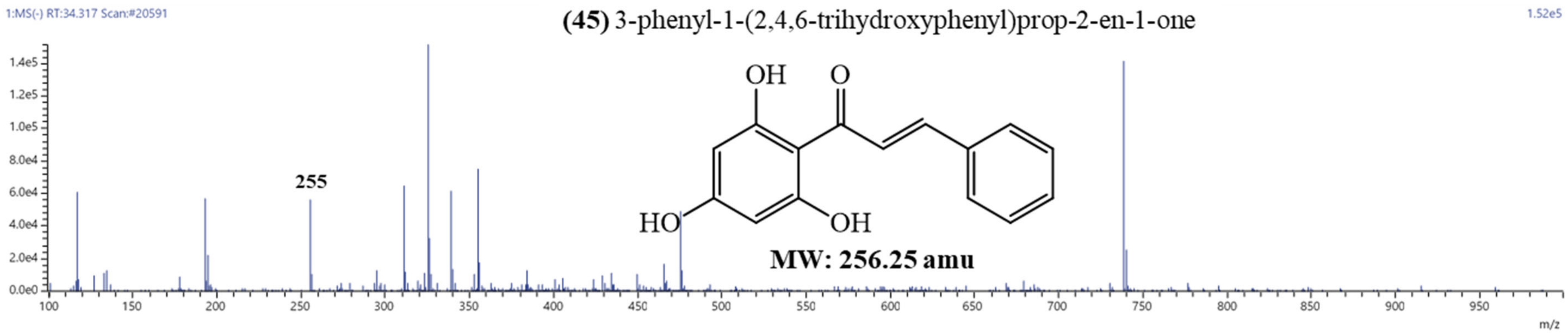

(46) Agestricin C (6-hydroxy-5,7,3',4'-tetramethoxyflavanone)

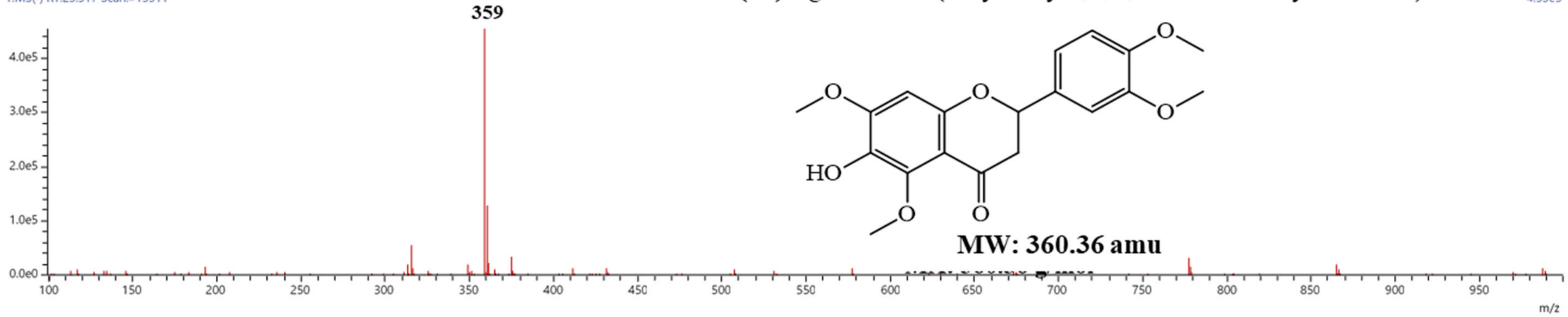

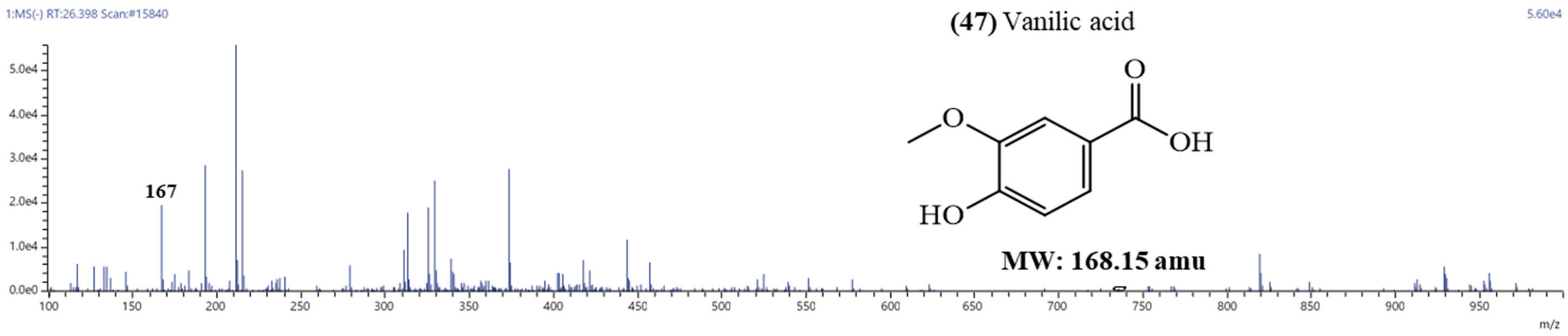
1:MS(-) RT:26.398 Scan:#15840
5.60e4
(47) Vanilic acid
167
O
OH
HO
MW: 168.15 amu
m/z

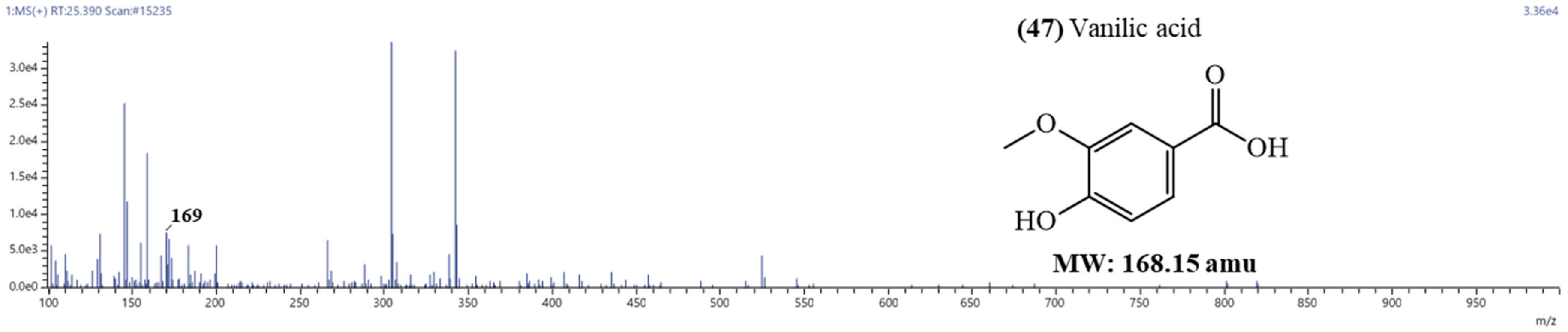
1:MS(+) RT:25.390 Scan:#15235
3.36e4
(47) Vanilic acid
169
O
OH
HO
MW: 168.15 amu
m/z

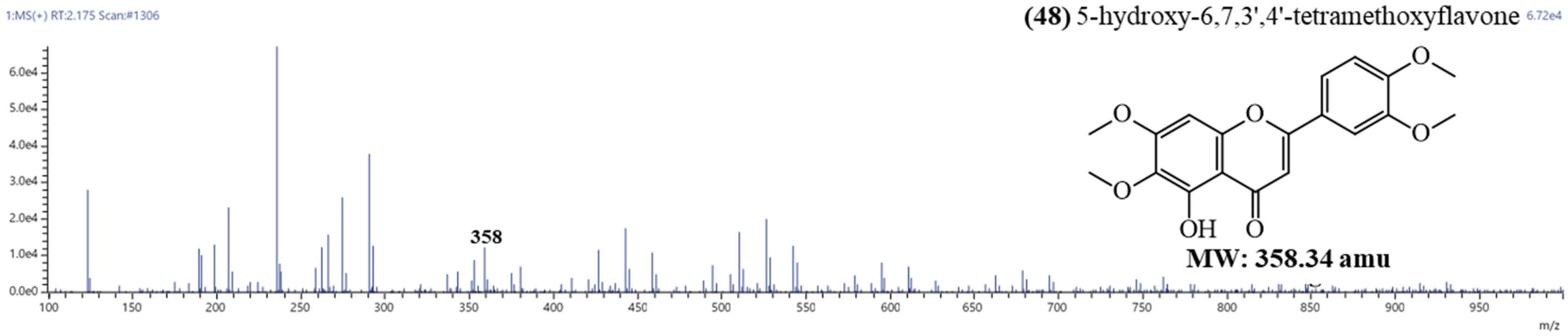
1:MS(+) RT:2.175 Scan:#1306
(48) 5-hydroxy-6,7,3',4'-tetramethoxyflavone
6.72e4
358
MW: 358.34 amu
m/z

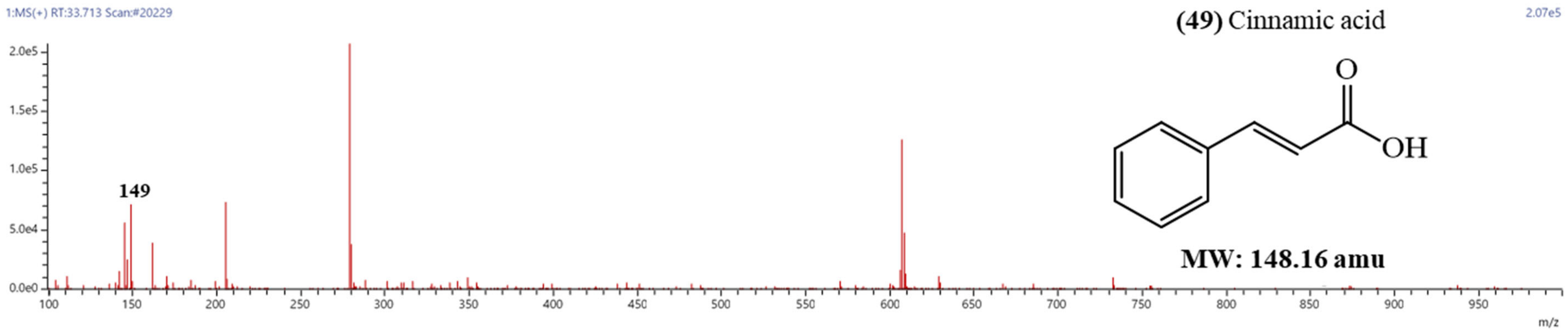
1:MS(+) RT:33.713 Scan:#20229
(49) Cinnamic acid
2.07e5
149
MW: 148.16 amu
m/z

(50) Nevadensin (5,7-dihydroxy-6,8,4'-trimethoxyflavone)

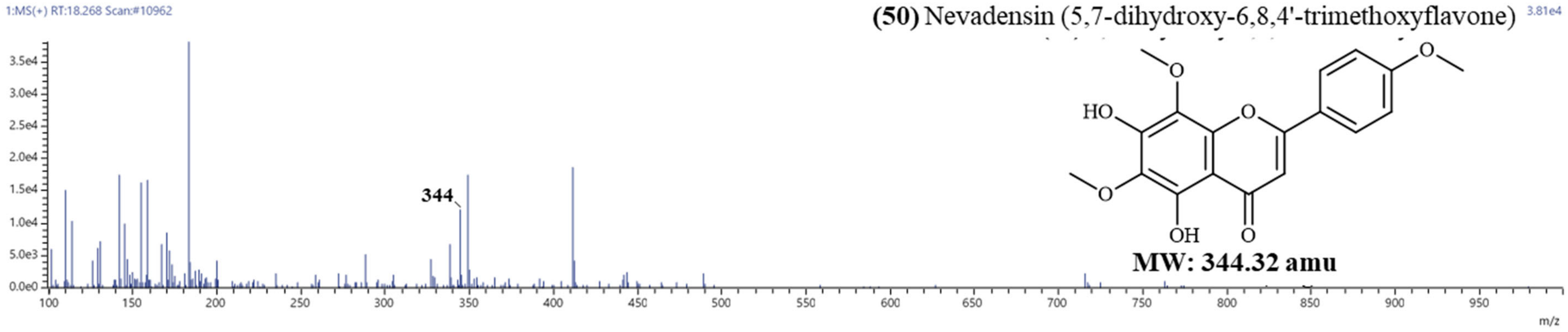

(51) Hesperitin (hesperetin-7-rutinoside)

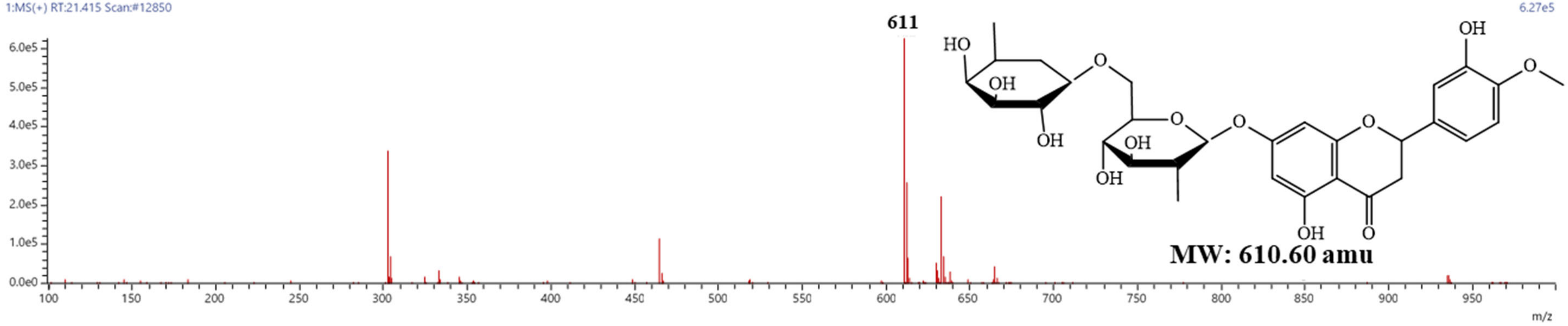

(52) Quercetin-3-O-α-L-rhamnopyranosyl-(1-6)-β-D-galactopyranoside

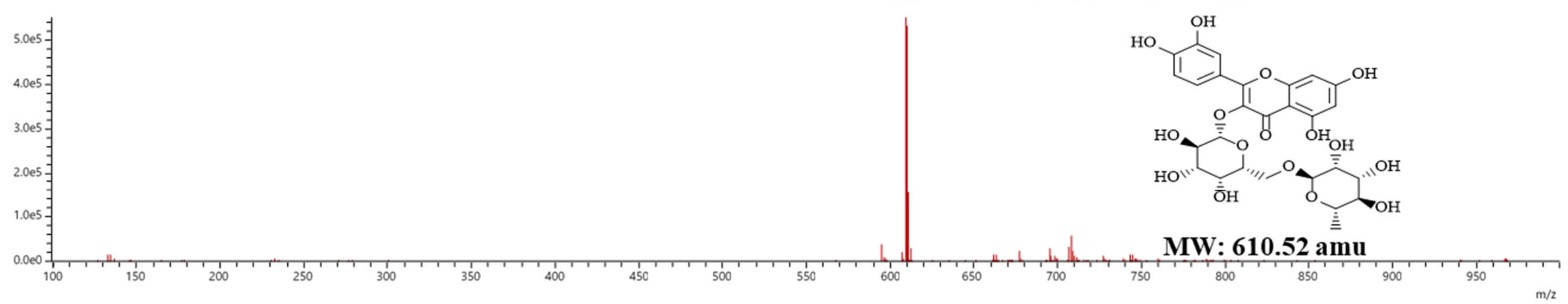

(53) Vicenin-2 (apigenin-6,8-di-C-glucoside)

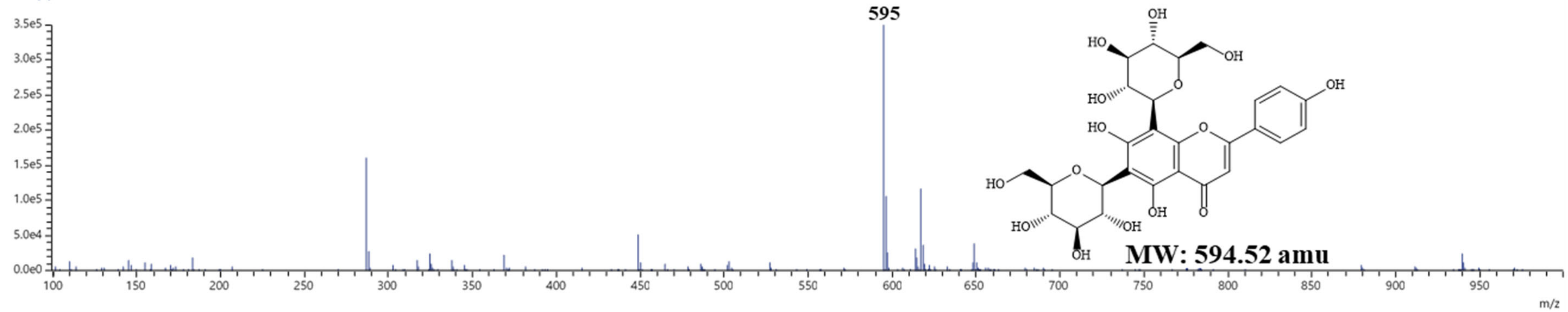

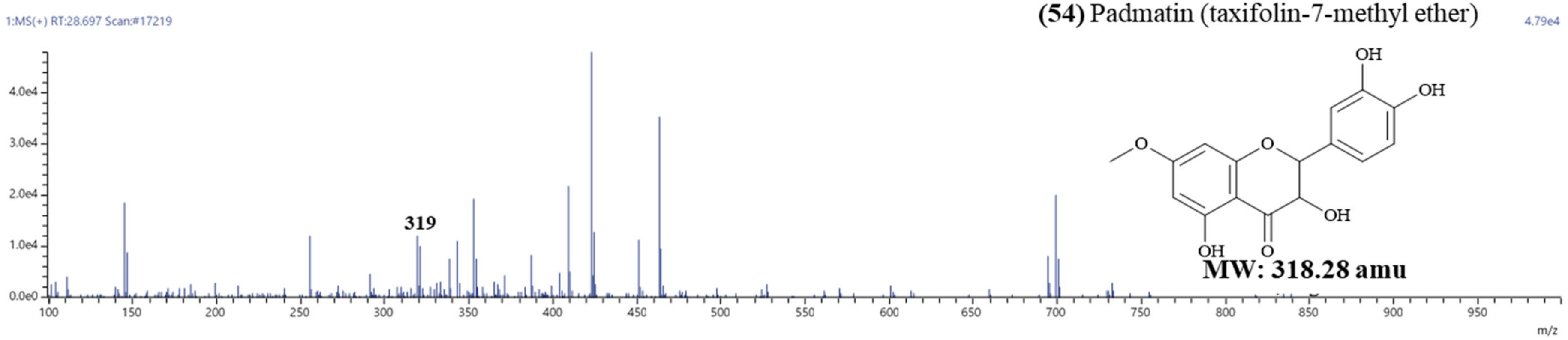

(54) Padmatin (taxifolin-7-methyl ether)

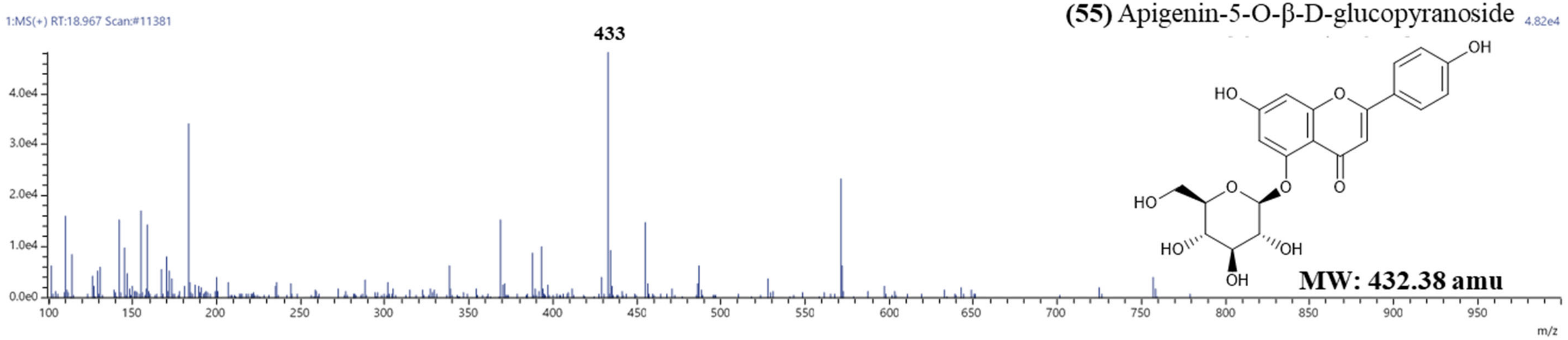

(55) Apigenin-5-O-β-D-glucopyranoside

(55) Apigenin-5-O-β-D-glucopyranoside

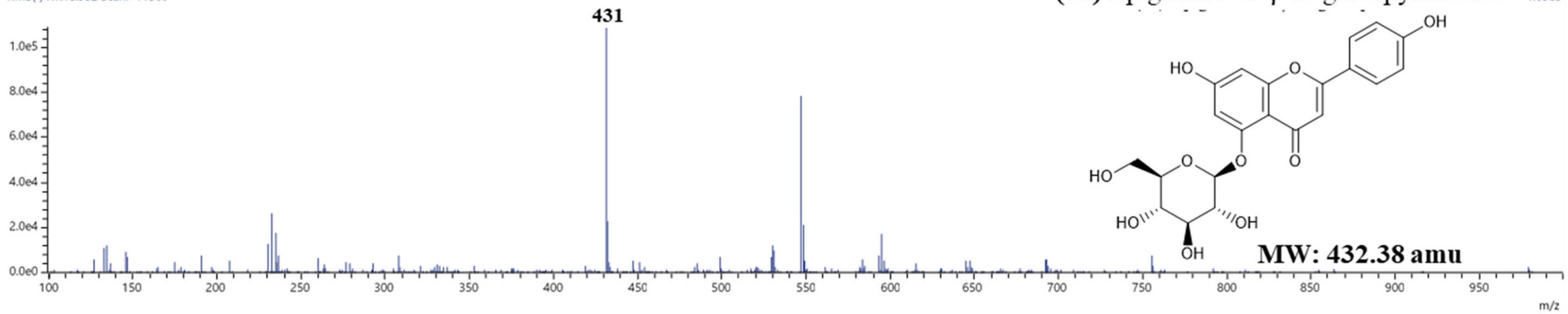

(56) Quercetin-3-O-β-D-glucuronide

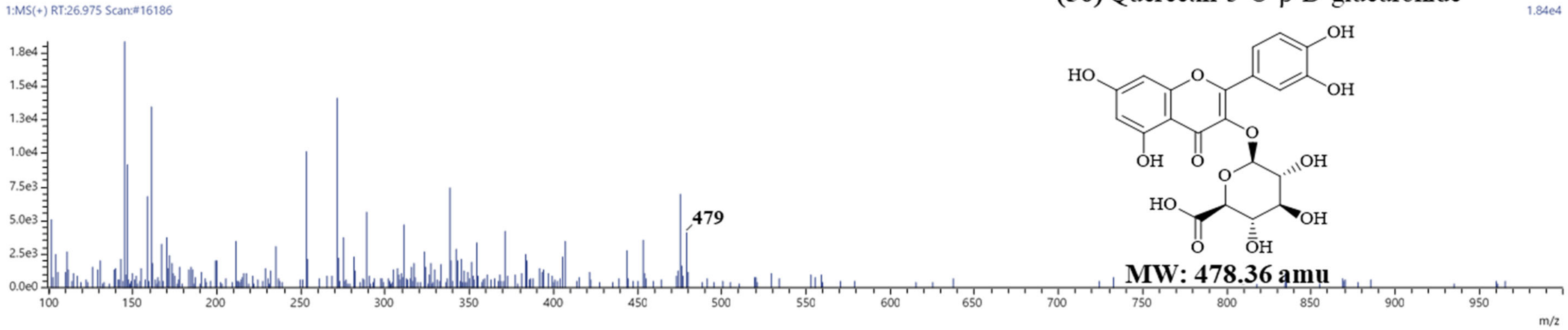

(57) Narcissin (isorhamnetin-3-rutinoside)

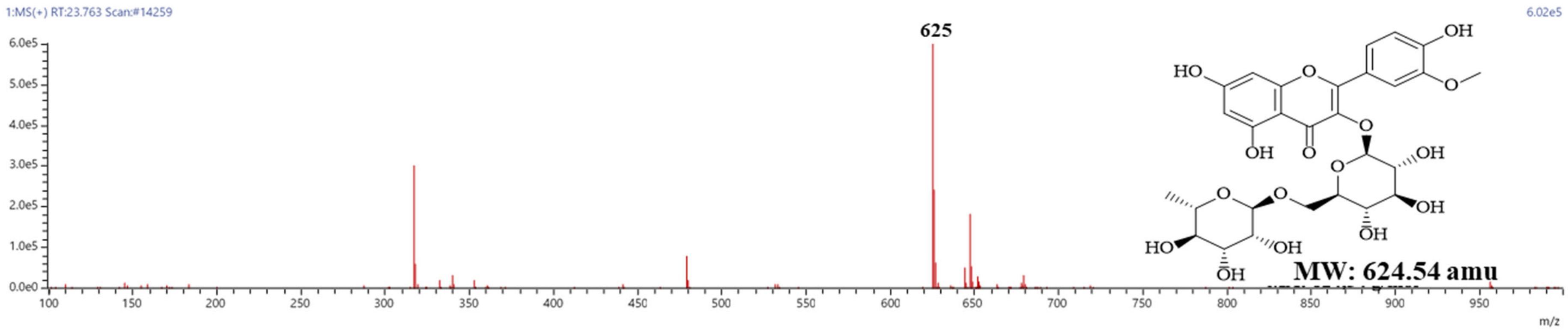

(57) Narcissin (isorhamnetin-3-rutinoside)

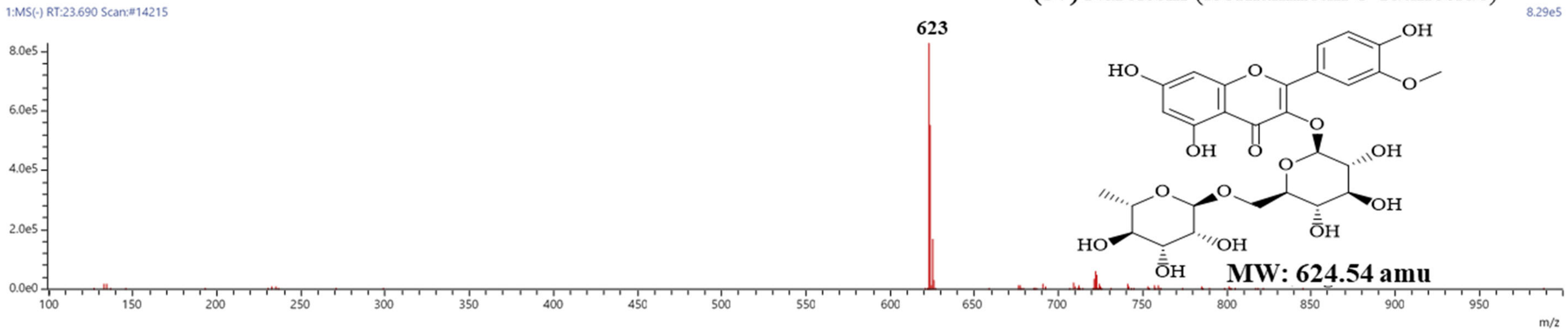

(58) Quercetin 3-O-α-L-arabinoside

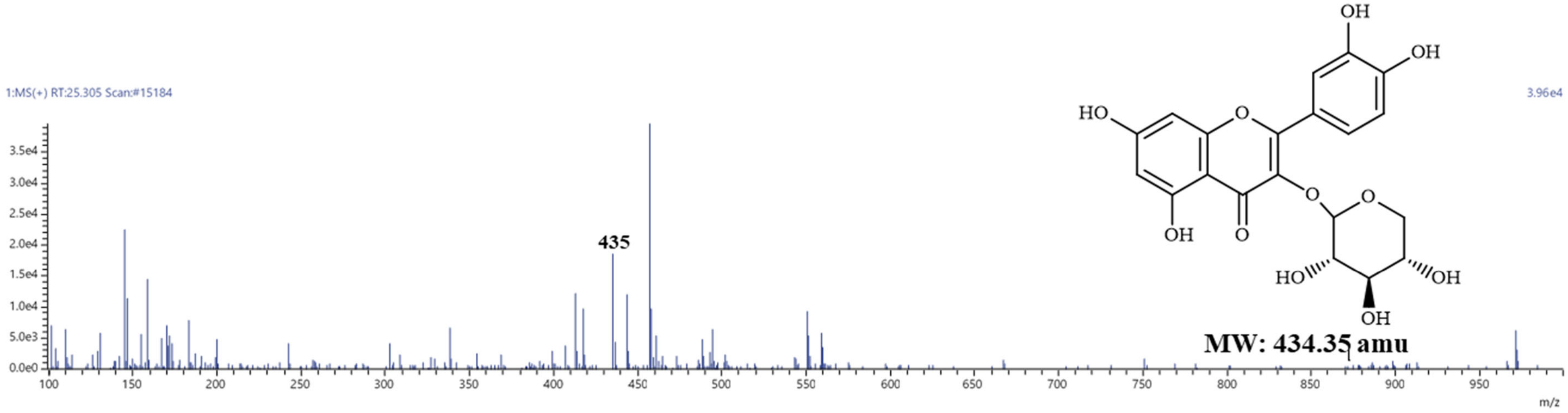

(58) Quercetin 3-O-α-L-arabinoside

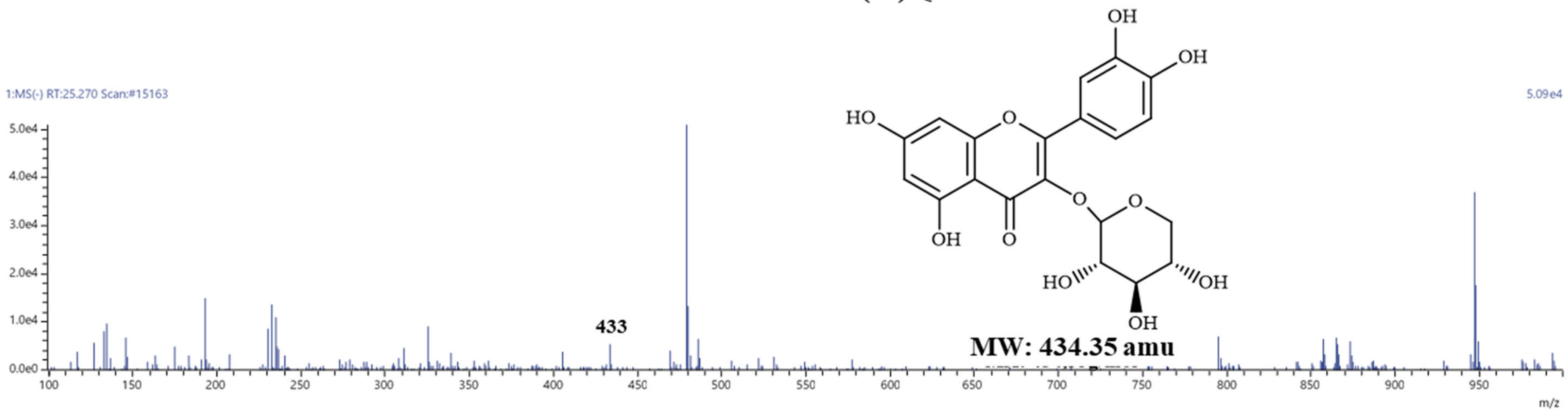

(59) Quercetin-3-O-rutinoside-7-O-glycoside

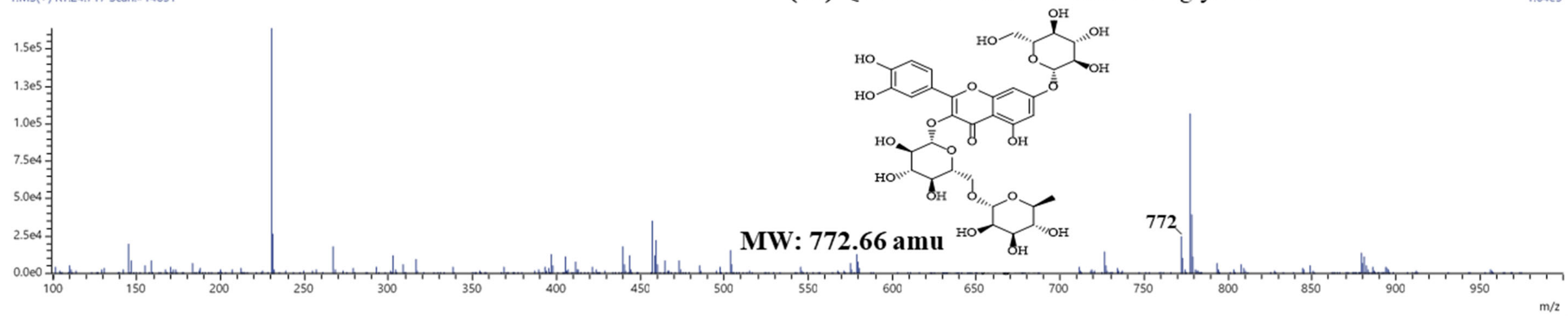

(59) Quercetin-3-O-rutinoside-7-O-glycoside

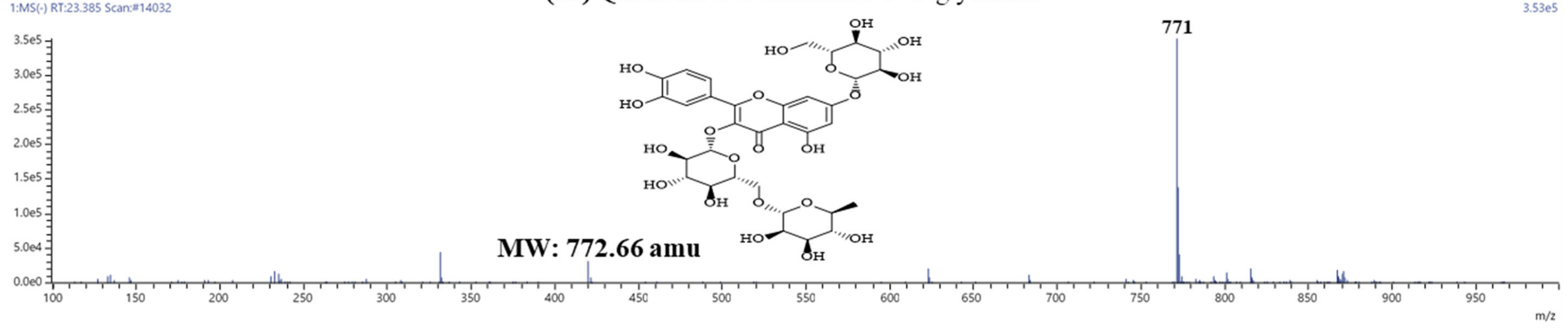

(60) Spireasalicin (Kuersetin-3-O-[6″-(4‴-hidroksi-2‴ -metilenbutirol)]- β-D-glukopiranosid)

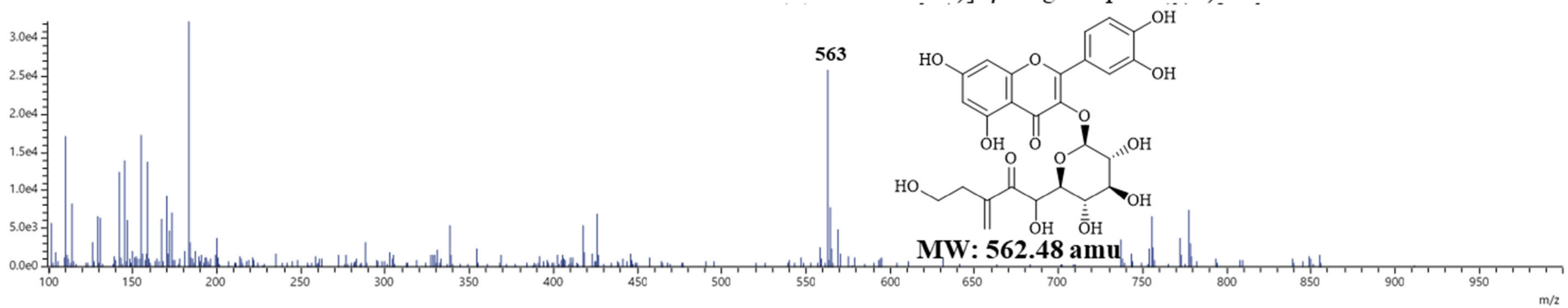

(61) (+)-Catechin-7-α-L-arabinofuranoside

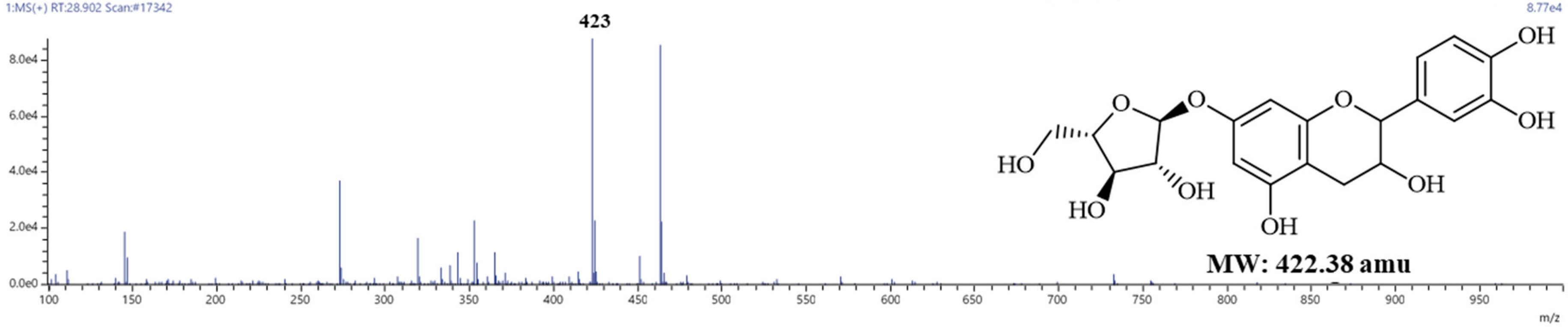

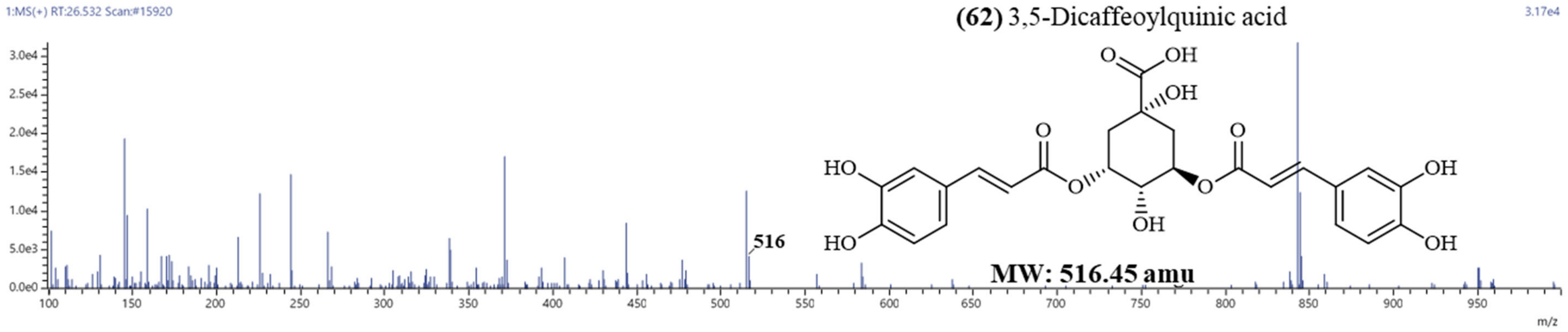
1:MS(+) RT:26.532 Scan:#15920
3.17e4
(62) 3,5-Dicaffeoylquinic acid
MW: 516.45 amu
516
m/z

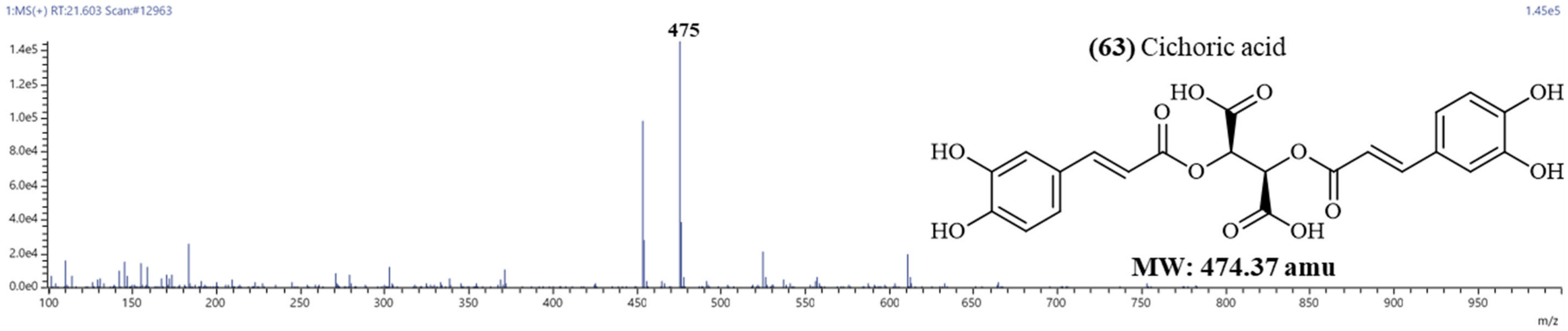
1:MS(+) RT:21.603 Scan:#12963
1.45e5
(63) Cichoric acid
MW: 474.37 amu
475
m/z

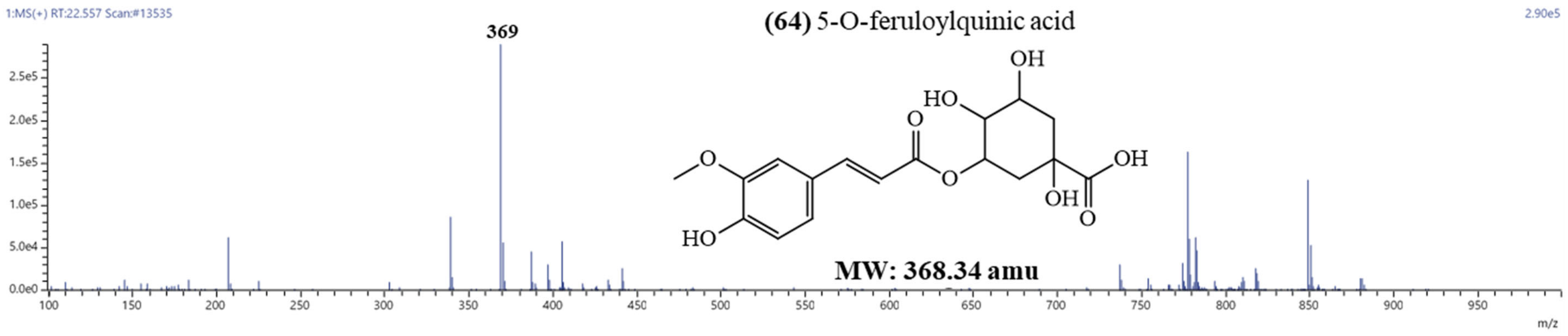
1:MS(+) RT:22.557 Scan:#13535
2.90e5
(64) 5-O-feruloylquinic acid
369
MW: 368.34 amu
m/z

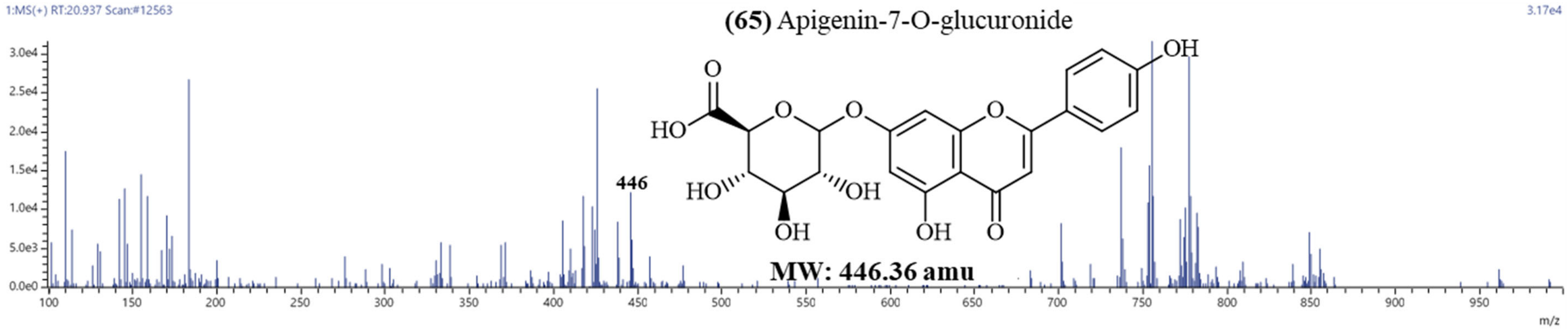
1:MS(+) RT:20.937 Scan:#12563
3.17e4
(65) Apigenin-7-O-glucuronide
446
MW: 446.36 amu
m/z

(66) Amentoflavone

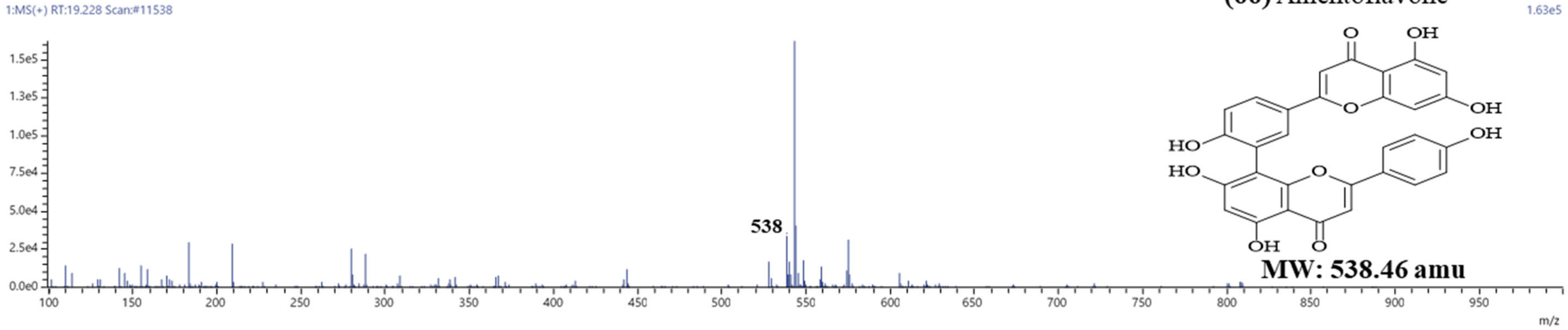

(67) Hiperosit (Quercetin-3-galactoside)

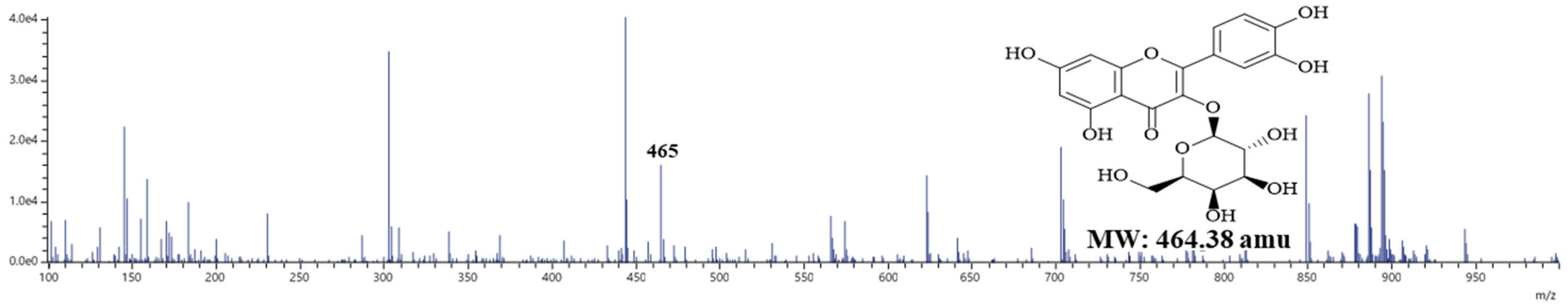

Supplement: Supplementary file 1 [file antioxidants-14-00153-s001.zip › antioxidants-3406948-supplementary.pdf]
